# Highly Soluble Dacarbazine Multicomponent Crystals Less Prone to Photodegradation

**DOI:** 10.1021/acs.molpharmaceut.4c00393

**Published:** 2024-06-10

**Authors:** Luan F. Diniz, Paulo S. Carvalho, Mateus A. C. Souza, Renata Diniz, Christian Fernandes

**Affiliations:** † Laboratório de Controle de Qualidade de Medicamentos e Cosméticos, Departamento de Produtos Farmacêuticos, Faculdade de Farmácia, Universidade Federal de Minas Gerais, 31270-901 Belo Horizonte, MG, Brazil; ‡ Departamento de Química, Instituto de Ciências Exatas (ICEx), Universidade Federal de Minas Gerais, 31270-901 Belo Horizonte, MG, Brazil; § Instituto de Física, Universidade Federal do Mato Grosso do Sul, 79074-460 Campo Grande, MS, Brazil

**Keywords:** dacarbazine, salt, cocrystal, cocrystallization, solubility, stability

## Abstract

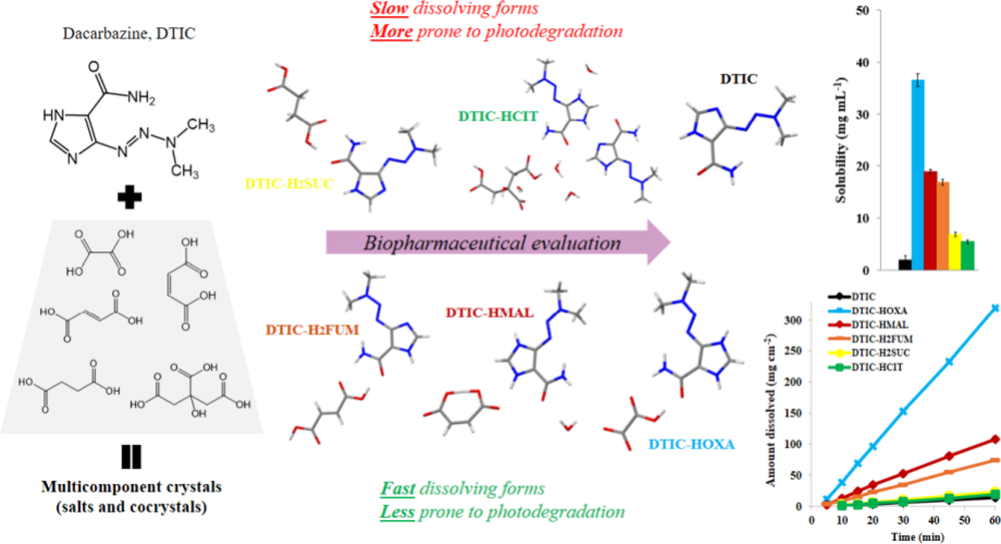

Dacarbazine (DTIC) is a widely prescribed oncolytic agent to treat advanced malignant melanomas. Nevertheless, the drug is known for exhibiting low and pH-dependent solubility, in addition to being photosensitive. These features imply the formation of the inactive photodegradation product 2-azahypoxanthine (2-AZA) during pharmaceutical manufacturing and even drug administration. We have focused on developing novel DTIC salt/cocrystal forms with enhanced solubility and dissolution behaviors to overcome or minimize this undesirable biopharmaceutical profile. By cocrystallization techniques, two salts, two cocrystals, and one salt-cocrystal have been successfully prepared through reactions with aliphatic carboxylic acids. A detailed structural study of these new multicomponent crystals was conducted using X-ray diffraction (SCXRD, PXRD), spectroscopic (FT-IR and ^1^H NMR), and thermal (TG and DSC) analyses. Most DTIC crystal forms reported display substantial enhancements in solubility (up to 19-fold), with faster intrinsic dissolution rates (from 1.3 to 22-fold), contributing positively to reducing the photodegradation of DTIC in solution. These findings reinforce the potential of these new solid forms to enhance the limited DTIC biopharmaceutical profile.

## 1 Introduction

**Scheme 1:**
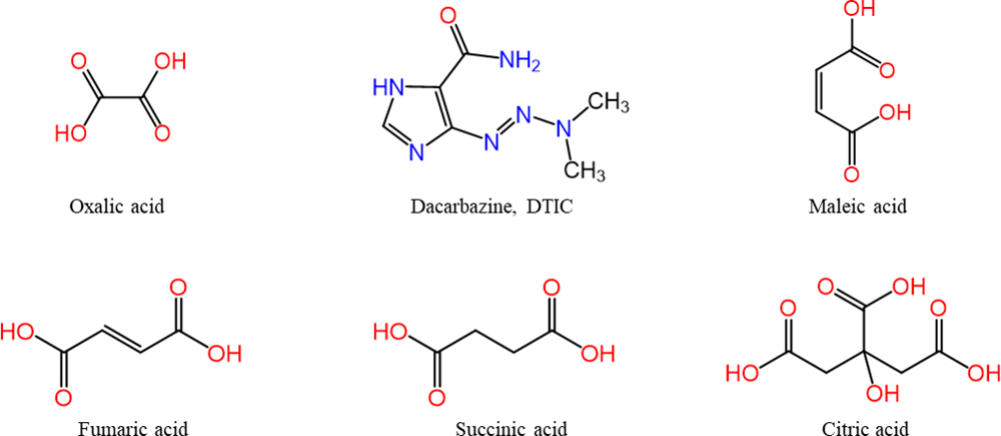
Molecular Structure of Dacarbazine (DTIC) and the Coformers: Oxalic, Maleic, Fumaric, Succinic, and Citric Acids

Crystal engineering is a powerful and well-established technology for generating improved multicomponent crystals, e.g., salts and cocrystals.^1−3^ Its extensive application in developing innovative active pharmaceutical ingredients (APIs) and the reform of older ones has made this technology receive widespread and unanimous acceptance from academia, industry, and drug regulatory agencies.^4−7^ Assembling custom-made crystalline arrangements by including API-complementary molecules (coformers) within the crystal lattice without breaking covalent bonds ensures the drug’s pharmacological activity while offering a pathway for fine-tuning its physicochemical properties (aqueous solubility, dissolution rate, solid-state stability, and hygroscopicity).^8−11^ Salt and cocrystal formation is the most effective and low-cost method employed in API screening/selecting steps.^12−14^ Usually, carboxylic acids recognized as safe (GRAS) are used as coformers to interact with weak basic drugs,^15^ such as dacarbazine (DTIC, Scheme 1), through hydrogen-bonding, converting it into salts or cocrystals.

DTIC is an anticancer drug indicated for the treatment of metastatic malignant melanoma and soft tissue sarcoma, being also used in the treatment of early Hodgkin lymphoma.^16,17^ Although it may be ionized, this API is commercialized in a neutral form as an intravenous formulation, exhibiting pH-dependent aqueous solubility and a pronounced photodegradation when exposed to light.^18,19^ The main photodegradation product, 2-azahypoxanthine (2-AZA), is pharmacologically inactive and further responsible for some adverse reactions.^20,21^ To reduce photodegradation, freshly prepared DTIC solutions protected from light must be intravenously administered as quickly as possible.^22,23^ Recently, Uchida et al.^24^ developed a photochemically stabilized formulation of DTIC using reactive oxygen species. The lack of scientific reports on this line reinforces the idea that the emergence of alternative and safe technologies to overcome DTIC issues remains highly demanded.

In the present work, we employ, for the first time, the crystal engineering strategy for the preparation of novel DTIC salt/cocrystal forms to elucidate how far cocrystallization can fine-tune the drug solubility and photostability. Through the proposition of a supramolecular synthesis protocol followed by an in-depth biopharmaceutical assessment, we introduce five novel multicomponent crystals of DTIC obtained from the drug’s reaction with pharmaceutically acceptable oxalic, maleic, fumaric, succinic, and citric carboxylic acids (Scheme 1). These novel solid forms have been characterized by single-crystal and powder X-ray diffraction (SCXRD and PXRD), Fourier transform infrared (FT-IR), proton nuclear magnetic resonance (^1^H NMR), thermogravimetry (TG), and differential scanning calorimetry (DSC). Equilibrium solubility, intrinsic dissolution, and photostability experiments were also carried out. All outcomes presented certainly insert valuable insights concerning improved DTIC manufacturing practices and drug administration, since their novel salts and cocrystals are biopharmaceutically advantageous over commercial DTIC.

## 2 Materials and Methods

### 2.1 Materials

Chromatographic-grade solvents were acquired commercially and used without further purification. All coformers (oxalic, maleic, fumaric, succinic, and citric acids) and dacarbazine were purchased from Sigma-Aldrich and used as-received. The 2-azahypoxanthine reference standard was purchased from the European Pharmacopoeia. Ultrapure water was obtained from a Millipore Direct-Q 3 UV system and used directly.

### 2.2 Preparation of DTIC Crystal Forms

#### 2.2.1 Dacarbazine Hydrogen-Oxalate Salt (DTIC-HOXA)

50 mg of DTIC (0.274 mmol) and 24.7 mg of oxalic acid (0.274 mmol) were manually ground in a pestle mortar by adding 1 mL of hot methanol until a homogeneous mass was formed. The resulting ground material was dissolved again in 5 mL of hot methanol for recrystallization. Suitable colorless prismatic crystals were observed within 1–2 days by slow evaporation of the solvent at room temperature.

#### 2.2.2 Dacarbazine Hydrogen-Maleate Salt (DTIC-HMAL)

50 mg of DTIC (0.274 mmol) and 31.8 mg of maleic acid (0.274 mmol) were mechanochemically reacted in a pestle mortar by adding 1 mL of a hot methanol/water (1:1, v/v) mixture until a homogeneous system was formed. Good-quality prismatic crystals of DTIC-HMAL appeared within 1–2 days by recrystallizing this material in 5 mL of hot methanol, maintained under slow evaporation at room temperature.

#### 2.2.3 Dacarbazine-Fumaric Acid Cocrystal (DTIC-H_2_FUM)

50 mg of DTIC (0.274 mmol) and 31.8 mg of fumaric acid (0.274 mmol) were macerated together in a pestle mortar by adding 1 mL of hot methanol until a homogeneous mass appeared. The ground material formed was dissolved in 5 mL of hot methanol for recrystallization using slow evaporation. Colorless block crystals were obtained within 2–3 days.

#### 2.2.4 Dacarbazine-Succinic Acid Cocrystal (DTIC-H_2_SUC)

50 mg of DTIC (0.274 mmol) and 32.4 mg of succinic acid (0.274 mmol) were mechanochemically reacted in a pestle mortar by adding 1 mL of hot methanol until a homogeneous mass was yielded. Suitable plate crystals of DTIC-H_2_SUC grew within 1–2 days by recrystallizing this material in 5 mL of hot methanol, which was kept under slow evaporation at room temperature.

#### 2.2.5 Dacarbazine Hydrogen-Citrate Salt-Cocrystal (DTIC-HCIT)

50 mg of DTIC (0.274 mmol) was weighed and dissolved in 5 mL of methanol/water (1:1, v/v) solution. Then, 52.7 mg (0.274 mmol) of citric acid was added, followed by stirring of the system at 60 °C for 20 min. Colorless block crystals were obtained within 7 days upon slow solvent evaporation at room temperature.

### 2.3 X-ray Diffraction Analysis

The single-crystal X-ray diffraction (SCXRD) data of the DTIC crystal forms were collected at room temperature (298 ± 2 K) in a Rigaku XtaLAB Synergy diffractometer with a HyPix detector and equipped with a Cu (λ = 1.54187 Å) microfocus radiation source. CrysAlisPro software was used for acquisition, indexing, integration, and unit cell determination. Subsequently, using Olex2,^25^ the structures were solved by intrinsic phasing with SHELXT^26^ and refined by full-matrix least-squares minimization with SHELXL.^27^ All non-hydrogen atoms were refined anisotropically. The hydrogen atoms, except those of the DTIC-HCIT structure, were located from electron-density difference maps, positioned geometrically, and refined freely. For DTIC-HCIT, the H atoms were also found from the electron-density difference map, but then they were geometrically refined using the riding model [*U*_iso_(H) = 1.2*U*_eq_ or 1.5*U*_eq_]. The visualization of structures and graphic material preparation has been made by MERCURY 4.3.1^28^ and ORTEP3^29^ for Windows programs. All CIF files are available at www.ccdc.cam.ac.uk under the CCDC^30^ numbers 2334988–2334992.

Powder X-ray diffraction (PXRD) measurements were carried out on an Empyrean PANalytical diffractometer at room temperature operating at 45 kV of voltage and 40 mA of current, in a Bragg–Brentano geometry, using CuKα radiation (λ = 1.54187 Å), Ni filter, and PIXcel3D detector. The diffractograms were acquired over an angular range of 2–50° (2θ) with a step size of 0.02° and a constant counting time of 4 s per step.

### 2.4 Spectroscopy Analysis

Fourier transform infrared (FT-IR) spectra were obtained on a PerkinElmer Spectrum One spectrometer equipped with an attenuated total reflectance (ATR) accessory in the 4000–650 cm^–1^ range with an average of 64 scans and a spectral resolution of 4 cm^–1^.

The ^1^H NMR data were acquired at room temperature on a Bruker Avance NEO spectrometer operating at 600 MHz, spectrum resolution of 0.12 Hz, zg30 pulse program with ns 16, d1 1s, acquisition time 4.09 s, and spectral width 20 ppm. Using Bruker’s TopSpin 4.0 software package, all ^1^H NMR spectra have been recorded and processed. The phase and baseline were manually corrected, and the TMS signals were calibrated at 0.00 ppm. Besides, integration regions of the signals were selected manually. Proton chemical shifts (δ) were given in ppm and coupling constants (*J*) in Hz. For the NMR experiments, 10 mg of each DTIC crystal form was dissolved in 600 μL of DMSO-*d*_6_ and transferred to NMR tubes for data acquisition.

### 2.5 Thermal Analysis

Thermogravimetric (TG) experiments were performed on a Shimadzu DTG-60 thermobalance, placing approximately 2.0 ± 0.2 mg of each sample in alumina pans and then heating at 10 °C min^–1^ under a nitrogen flow (50 mL min^–1^) from 25 to 600 °C. Differential scanning calorimetry (DSC) curves were obtained using a Shimadzu DSC-60 instrument. The samples (1.0 ± 0.2 mg) were placed in aluminum pans and heated at a constant rate of 10 °C min^–1^ under a nitrogen flow (50 mL min^–1^). According to TG data, the DSC measurements have been conducted until the degradation temperature of each compound. Shimadzu TA-60 software was employed in the data analysis.

### 2.6 Liquid Chromatography and Mass Spectrometry Conditions

The quantitative analyses of DTIC and 2-AZA were performed by liquid chromatography (LC) with UV detection (LC-UV) in a Waters ACQUITY UPLC system equipped with an ultraviolet detector. Empower 3.0 software was employed for data acquisition and analysis. A Waters CORTECS C18 column (150 mm length × 4.6 mm i.d., 2.7 μm particle size), maintained at 40 °C, was used for separation. The mobile phase consisted of a mixture of 0.1% (v/v) aqueous formic acid solution (A) and acetonitrile (B), in a gradient elution mode (0.0–2.5 min: 0% B; 2.5–4.0 min: 0–6% B; 4.0–5.0 min: 6–0% B; and 5.0–7.0 min: 0% B), delivered at a flow rate of 0.8 mL min^–1^. DTIC and 2-AZA were detected at 240 nm, and the injection volume was 5 μL. The method validation followed the ICH Q2 (R2) guideline.^31^ The parameters selectivity, linearity, precision, accuracy, and limits of quantitation and detection were evaluated. The main results found are summarized in Tables S1 and S2.

Liquid chromatography–tandem mass spectrometry (LC-MS/MS) experiments were performed on an Agilent 1200 system coupled to a Sciex QTRAP 5500 mass spectrometer equipped with a Turbo IonSpray ionization source in the electrospray mode. The software Analyst ver. 1.6.2 was used for data acquisition and analysis. Initially, the presence of DTIC and 2-AZA precursor ions in a photodegraded solution (see Section 2.8) was confirmed by direct infusion in a full scan mode, from 50 *m*/*z* 50 to 200. Then, 5 μL of the degraded solution was injected into the LC-MS/MS system, employing the same conditions used in the LC-UV analysis. For mass spectrometry detection, a product-ion monitoring scan mode was used. The precursor ions of DTIC (*m*/*z* 183) and 2-AZA (*m*/*z* 138) have been monitored (declustering potentials of 146 V to DTIC and 86 V to 2-AZA) and fragmented (collision energies of 47 V to DTIC and 20 V to 2-AZA; collision cell exit potential of 10 V) to obtain their product ions. The conditions of the electrospray ionization source were set as follows: ion-spray voltage, 5 kV; collision-activated dissociation, medium; gas temperature, 750 °C; curtain gas, 15 psi; nebulizing gas, 50 psi; and auxiliary gas, 50 psi. Nitrogen was used as both a nebulizing and desolvation gas.

### 2.7 Equilibrium Solubility and Intrinsic Dissolution

Equilibrium solubility values of DTIC crystal forms were stated by the shake-flask method^32^ at 37.0 ± 0.5 °C in buffered aqueous media with pH ranging from 1.2 to 6.8. The dissolution media preparations, i.e., HCl solution pH 1.2, acetate buffer pH 4.5, and phosphate buffer pH 6.8, are listed in Table S3. Suspensions, in triplicate, were prepared by stirring an excess amount of solid, sufficient to reach saturation, into 2 mL of each dissolution medium for 24 h. Then, these suspensions were filtered through a 0.45 μm syringe filter and diluted in their respective dissolution media before being quantified by LC-UV. After the equilibrium solubility experiments, the solid sediment identity was checked by PXRD analysis, and the pH value in each dissolution medium was measured by using a pH meter.

Intrinsic dissolution tests were carried out in triplicate using a rotating disk dissolution apparatus. Approximately 200 mg of each DTIC crystal form was compressed by a hydraulic pump at 1 kN for 1 min to form nonporous and compact 0.5 cm^2^ disks with a flat surface. The attachments containing the disks were immersed into 500 mL of phosphate buffer, pH 6.8, medium preheated at 37.0 ± 0.5 °C with the metallic rod rotating at 100 rpm. At specific time intervals (5, 10, 15, 20, 30, 45, and 60 min), 5 mL of dissolution medium was withdrawn and immediately filtered through a 0.45 μm syringe filter before the quantification of dissolved DTIC by LC-UV.

### 2.8 Stability Studies

First, all DTIC crystal forms were exposed under accelerated degradation conditions, specifically at 40 °C and 75% relative humidity (RH), for 90 days to assess the drug’s solid-state stability. Each solid was stored in an open Petri dish before being placed inside a desiccator containing a saturated sodium chloride solution (to generate 75% RH). Subsequently, the desiccator was maintained in a temperature-controlled oven at 40 °C. A thermohygrometer, kept with the samples, proved that temperature and relative humidity remained constant over the 90 days. The solid-state stability of the new DTIC forms was also checked through photodegradation tests. For this, a dark woody chamber equipped with a black UV lamp (300 mm, 15 W) was used. The DTIC crystals were placed 30 cm from the light source in open Petri dishes for 15 days. In both studies, the DTIC content was verified by LC-UV.

Finally, a photostability experiment in aqueous media was performed to evaluate the degradation kinetics of DTIC and formation of photoproduct 2-AZA by dissolving the different DTIC crystal forms in 5 mL of phosphate buffer, pH 6.8. A triplicate of freshly prepared solutions at a concentration of 5 mM has been placed into clear glass vials and arranged at 30 cm from a black UV lamp (300 mm, 15 W) to be photoirradiated for 5 h. Hourly, the remaining DTIC and the amount of 2-AZA in the samples were determined using the validated LC-UV method described in the previous Section 2.6.

## 3 Result and Discussion

Overall, considerable improvements in aqueous solubility are reached by multicomponent crystals that display structures with a prevalence of hydrophilic domains over hydrophobic ones. This structural attribute aids the accessibility of water molecules during the dissolution process.^33^ Hence, DTIC salt/cocrystal formation from di- or tri carboxylic acids, inserting molecules rich in polar domains (coformers) into a more hydrophilic structure, stabilized by hydrogen bond, fits perfectly with this requirement. On the other hand, the ability of these same supramolecular systems to improve API photostability is not a straightforward task. There is little consensus in the literature regarding the structural attributes required for the engineered API to become less susceptible to photodegradation. We only know that the occurrence of strong and stabilizing intermolecular interactions in the crystal structure, enhancing and diversifying the occurrence of synthons, may be a prerequisite for photostability improvement.^34^ Indirectly, improvements in solubility imply faster pharmaceutical processes based on dissolution, minimizing hydrolysis and photodegradation reactions, justifying again the choice of aliphatic carboxylic acids as coformers.

An interesting insight derived from a work that investigated the photostabilizing effect of cysteine on the photodegradation of DTIC^24^ motivated us to employ amino acids as cocrystallization agents. We hypothesized that a multicomponent crystal composed of DTIC and biomolecules, known to be photoprotective for DTIC, could address the drug photostability issue. Nevertheless, the reactions and crystallization experiments between DTIC and several amino acids tested, including cysteine, have failed without any solid/crystal forming at the end.

DTIC is a weak base (p*K*_a_ = 4.4),^35^ and salt formation was expected when the API reacted with the two shorter-chain carboxylic acids considered relatively strong, i.e., oxalic and maleic acids. The p*K*_a_ difference (Δp*K*_a_) found for these reactions is close to 3 units (Table S4), suggesting salt formation.^36^ The difference between the p*K*_a_ values of DTIC and fumaric, succinic, and citric longer-chain carboxylic acids gives Δp*K*_a_ values within the range from 0 to 2 (Table S4). Within this interval, referred to as the continuum region, salt or cocrystal forms are equally likely to be obtained.^37^ Thus, the assessment of spectroscopic data (FT-IR and ^1^H NMR) combined with bond length evaluation (C–O distances) and electron density map interpretation (by SCXRD) proved each DTIC solid form’s ionic/nonionic nature.

### 3.1 Crystallographic Evaluation

A total of five multicomponent systems of DTIC were supramolecularly synthesized: two salts, the first anhydrous with oxalic acid (DTIC-HOXA) and the second monohydrated with maleic acid (DTIC-HMAL), two anhydrous cocrystals with fumaric and succinic acids (DTIC-H_2_FUM and DTIC-H_2_SUC, respectively), and finally a tetrahydrated salt-cocrystal with citric acid (DTIC-HCIT). Herein, the novel crystals have been prepared through traditional recrystallization and solvent evaporation techniques (see Section 2.2). To understand the structural aspects that govern the stabilization of these systems in the solid-state, crystallographic evaluations are provided below. The asymmetric units (ASUs) of the DTIC solid forms are shown in Figure S1. Tables 1 and S5 summarize the crystallographic data and H-bond geometric parameters, respectively. Figures S2–S4 display the electron density maps of each DTIC crystal form, confirming unequivocally the protonation and nonprotonation sites of the DTIC molecules, assigning whether salt or cocrystal has been formed.

**Table 1:** Crystallographic Data and Refinement Parameters of the DTIC Solid Forms

identification code	DTIC-HOXA	DTIC-HMAL	DTIC-H_2_FUM	DTIC-H_2_SUC	DTIC-HCIT
chemical formula	C_8_H_12_N_6_O_5_	C_10_H_16_N_6_O_6_	C_10_H_14_N_6_O_5_	C_10_H_16_N_6_O_5_	C_18_H_36_N_12_O_13_
molecular weight	272.24	316.29	298.27	300.29	628.59
temperature (K)	298(2)	298(2)	298(2)	298(2)	298(2)
crystal system	monoclinic	triclinic	monoclinic	monoclinic	monoclinic
space group	*P*2_1_/*n*	*P*1̅	*I*2/*m*	*P*2_1_/*n*	*P*2_1_
*a* (Å)	7.01610(10)	7.20630(10)	14.9598(4)	10.18050(10)	7.50230(10)
*b* (Å)	14.5257(2)	9.5417(2)	6.5349(2)	5.28880(10)	23.4227(5)
*c* (Å)	11.4853(2)	11.4641(2)	14.7476(3)	25.7639(4)	8.5781(2)
α (°)	90	111.919(2)	90	90	90
β (°)	100.475(2)	94.2000(10)	102.407(2)	90.9610(10)	103.211(2)
γ (°)	90	90.5110(10)	90	90	90
volume (Å)^3^	1151.00(3)	728.77(2)	1408.07(6)	1387.00(4)	1467.49(5)
*Z*/*Z*′	4/1	2/1	4/1	4/1	2/2
ρ_calc (_g cm^3^)	1.571	1.441	1.407	1.438	1.423
μ (mm^–1^)	1.142	1.035	0.985	1.000	1.045
radiation type	Cu *K*α	Cu *K*α	Cu *K*α	Cu *K*α	Cu *K*α
2θ range for data collection/°	9.92–140.11	10–139.98	9.50–140.14	9.29–140.04	10.6–140.11
reflections collected	11043	13712	7398	13467	10839
independent reflections	2175	2752	1453	2631	4953
unique reflections	1968	2508	1280	2392	4713
*R*_1_ [*I* ≥ 2σ(*I*)]	0.0365	0.0337	0.0409	0.0382	0.0463
*wR*_2_ [all data]	0.1073	0.0981	0.1257	0.1075	0.1252
goodness-of-fit on *F*^2^	1.018	1.085	1.062	1.058	1.063

The prediction of salt/cocrystal formation based on the p*K*_a_ rule^38^ and acid chain length^39^ was accurate. As expected, proton transfer from the acid to the base (DTIC) occurred when the Δp*K*_a_ of the reactions was superior to 2.5 units, as shown in Table S4. Not coincidentally, oxalic and maleic acids, which have slightly shorter carbon chain lengths, are the coformers most likely to contribute to salt formation. In the cocrystal setting, increasing the aliphatic chain length of the acid/coformer contributes to the nonoccurrence of proton transfer reactions. Indeed, we have achieved cocrystals from the reactions between DTIC and fumaric, succinic, and citric coformers (acids with slightly longer carbon chain lengths). Corroborating this, the resulting Δp*K*_a_ values between DTIC and these three coformers are less than 1.5 units (Table S4). According to the Δp*K*_a_ rule, the occurrence of cocrystals notably increases when the Δp*K*_a_ values are less than 1.5 units, which is in line with our findings.

#### 3.1.1 Dacarbazine Hydrogen-Oxalate (DTIC-HOXA) Salt

**Figure 1:**
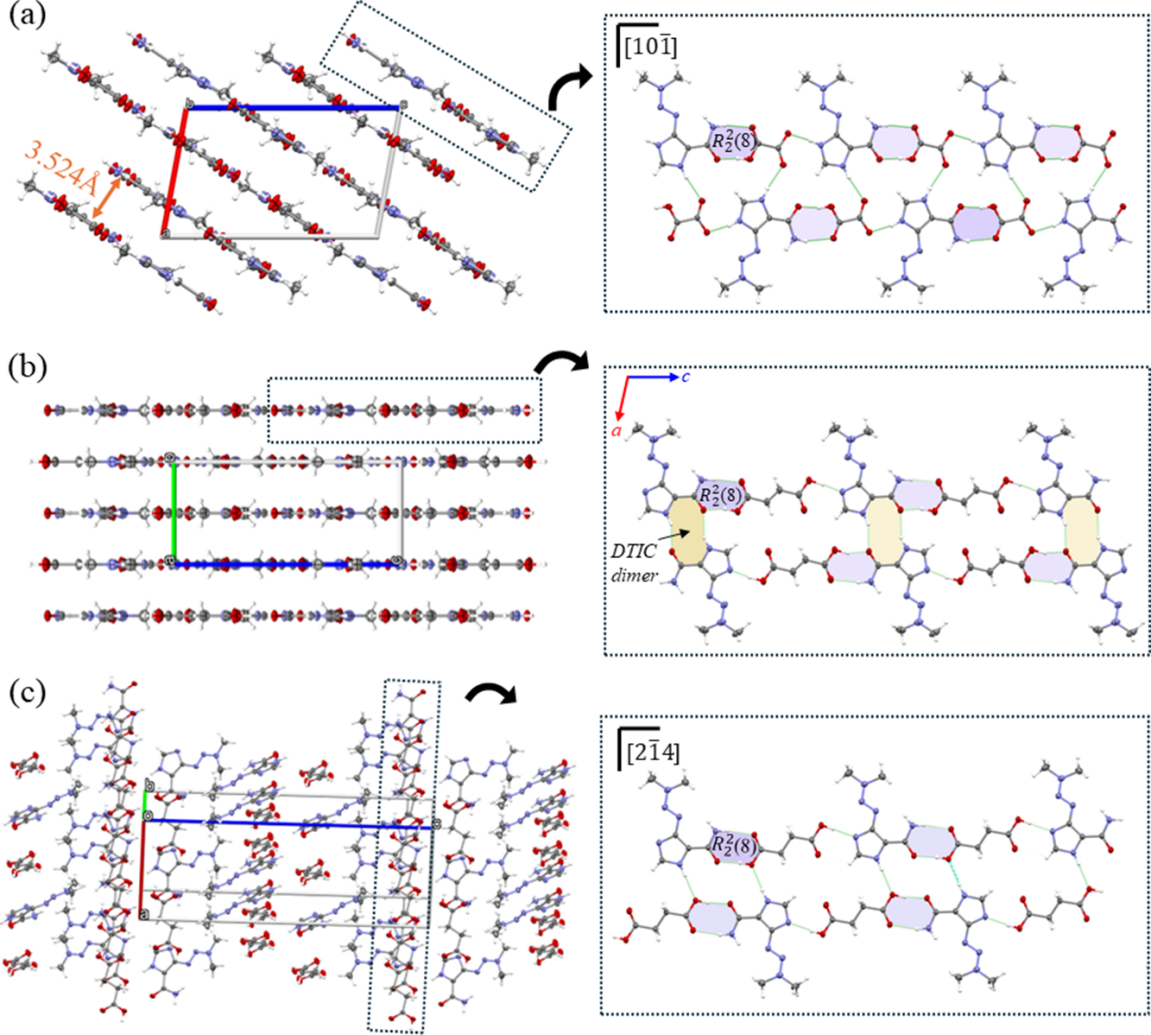
2D sheet motifs from the chain assemblies and layered crystalline packings for (a) DTIC-HOXA, (b) DTIC-H_2_FUM, and (c) DTIC-H_2_SUC.

The DTIC-HOXA is a 1:1 salt crystallizing in the monoclinic *P*2_1_/c space group with *Z*′ = 1, and its ASU (Figure S1) comprises a dacarbazinum (DTICH^+^) cation and a hydrogen-oxalate (HOXA^–^) anion. The DTIC-HOXA salt is formed by transferring one proton from one of the two carboxylic groups of oxalic acid to the DTIC imidazole ring. After protonation to form a salt, the anion presents a carboxylate group with two similar C–O distances and a carboxylic group with distinct C–O distances. Then, the ionic pair of salt is stabilized by a single N–H···O H-bond. The main supramolecular structure is a 1D chain of ionic pairs (Figure 1a). Along the [001] direction, they are held together by an *R*_2_^2^(8) synthon between amide and carboxylic groups. Along the chain, the cations are alternately extended with the anions, exposing the amide groups to the exterior of this motif. It allows two adjacent chains to form a 2D sheet. As expected from the planar geometry of the ion pairs, DTIC-HOXA consists of a layered structure. The 2D sheets are centrosymmetrically shifted and stacked with each other along the [101̅] direction, stabilized by C–H···O interactions between adjacent cations to form a 3D structure, as shown in Figure 1a.

#### 3.1.2 Dacarbazine-Fumaric Acid (DTIC-H_2_FUM) Cocrystal

Reacting DTIC and fumaric acid (H_2_FUM) yielded a 1:1 cocrystal, belonging to the monoclinic group *I*2/m with *Z*′ = 1. The ASU is characterized by a H_2_FUM and a neutral DTIC molecule (Figure S1). The cocrystal formation is confirmed by analyzing the bond length ratios of C–O of COOH in H_2_FUM and the electron density map (Figure S3). The average difference of 0.145 Å indicates that the acid structure is not derived from deprotonation. DTIC and H_2_FUM recognize each other, forming a 1D motif analogous to that observed in the oxalate salt. Along the [001] direction, a DTIC molecule connects to H_2_FUM via a single N–H···O H-bond. Besides, these units assembled to each other by *R*_2_^2^(8) synthon connects the adjacent unit carboxylic and imidazole groups. Due to the nonionization of APIs, adjacent chains are connected into a 2D sheet by a dimeric association of DTIC involving the imidazole and amide groups. Unlike the oxalate salt, 2D sheets align infinitely in the *ac* plane through C–H···O H-bonds between neighboring DTIC and H_2_FUM molecules. These plane motifs, in turn, stack centrosymmetrically to each other along the [010] direction via C–H···O interactions, resulting in a layered structure (Figure 1).

#### 3.1.3 Dacarbazine-Succinic Acid (DTIC-H_2_SUC) Cocrystal

Succinic acid (H_2_SUC) also forms cocrystals with DTIC (Figure S1). The DTIC-H_2_SUC cocrystal crystallized in the monoclinic *P*2_1_/c space group with *Z*′ = 1. Likewise, the dissimilarity of the carboxyl group’s C–O distances in H_2_SUC corroborated by electron density map analysis (Figure S3) proves the nonionization of DTIC in the DTIC-H_2_SUC structure. H_2_SUC and H_2_FUM differ only by the central portion saturation of the molecule such that H_2_SUC is associated with DTIC forming a 2D sheet motif with analogues found in the DTIC-H_2_FUM cocrystal (Figure 1c). However, DTIC-H_2_SUC and DTIC-H_2_FUM are not isomorphic cocrystals. In the DTIC-H_2_SUC cocrystal, the 2D sheet structures stack to form a columnar arrangement along the [21̅4] direction. Unlike the fumarate cocrystal, along the [001] direction, neighboring columns assemble almost orthogonally with each other and are alternately arranged along this direction, forming a 3D structure, as shown in Figure 1c.

#### 3.1.4 Dacarbazine Hydrogen-Maleate (DTIC-HMAL) Salt

**Figure 2:**
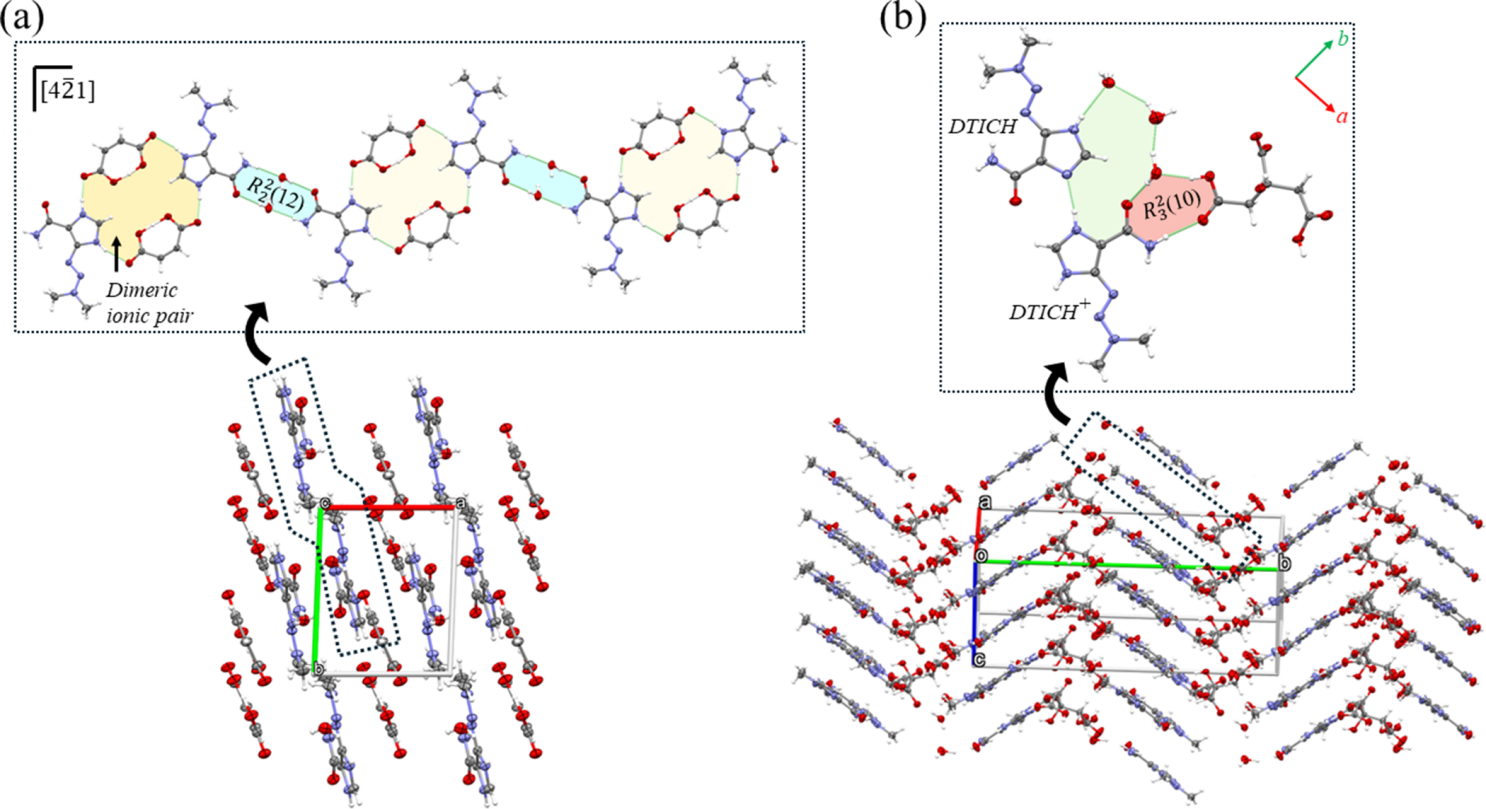
2D sheet arrangements of chains and layered crystalline assemblies for (a) DTIC-HMAL and (b) DTIC-HCIT.

Maleic acid is a *cis*-isomer of H_2_FUM and forms a hydrated salt with DTIC. After acid deprotonation, the COO^–^ of the anion exhibits two similar C–O distances, indicating the bond electronic resonance due to the ionization. DTIC-HMAL is a hydrated salt that crystallizes in the triclinic *P*1̅ space group having *Z*′ = 1. The ASU comprises a dacarbazinum (DTICH^+^) cation, a hydrogen-maleate anion (HMAL^–^), and a water molecule (Figure S1). Two ionic pairs form a dimeric unit in the structure through NH^+^_imidazol_···COO^–^ bonds (Figure 2a). In this unit, the HMAL^–^ anions connect two DTICH^+^ cations, arranging them centrosymmetrically. Like the other multicomponent crystals reported here, the ionic pairs recognize each other, generating structural motifs for stacking in molecular layers. The water molecules play an important structure-forming role acting as bridges between the ionic pairs through a *R*_2_^2^(12) ring synthon formed by the amide group of cations, two water molecules, and the COO^–^ groups of anions. Hence, a 2D sheet motif runs along the [42̅4] direction. Finally, a layered structure (Figure 2a) is formed as a packing result of these sheet motifs due to the presence of O_w_H···COO^–^ H-bonds.

#### 3.1.5 Dacarbazine Hydrogen-Citrate (DTIC-HCIT) Salt-Cocrystal

As citric acid is triprotic, its reaction in equimolar amounts with DTIC resulted in a hydrated salt-cocrystal. DTIC-HCIT crystallized in the monoclinic *P2*_*1*_ space group with *Z*′ = 2. The ASU contains a DTICH^+^ cation, a hydrogen-citrate anion (H_2_CIT^–^), a neutral DTIC, and four independent water molecules (Figure S1). In the salt-cocrystal structure, the DTICH^+^ and H_2_CIT^–^ ions do not display their ionizable groups directly H-bonded to each other. Still, they are stabilized by an *R*_3_^2^(10) synthon between both amide group and COO^–^ groups and a water molecule (Figure 2b). This system binds to neutral DTIC through a (H_2_O)_2_ cluster and N–H···O H-bond between the charged and neutral API molecules to form a structural cocrystal unit. These units are then stacked along the [001] direction in a columnar arrangement due to the incorporation of water molecules. Along the [010] direction, adjacent columns assembled each other due to COOH···H_2_O H-bonds orienting the columns in a zigzag fashion. Such columns are alternated with hydrophilic layers consisting of anions and water molecules (Figure 2b).

### 3.2 Powder X-ray Diffraction

**Figure 3:**
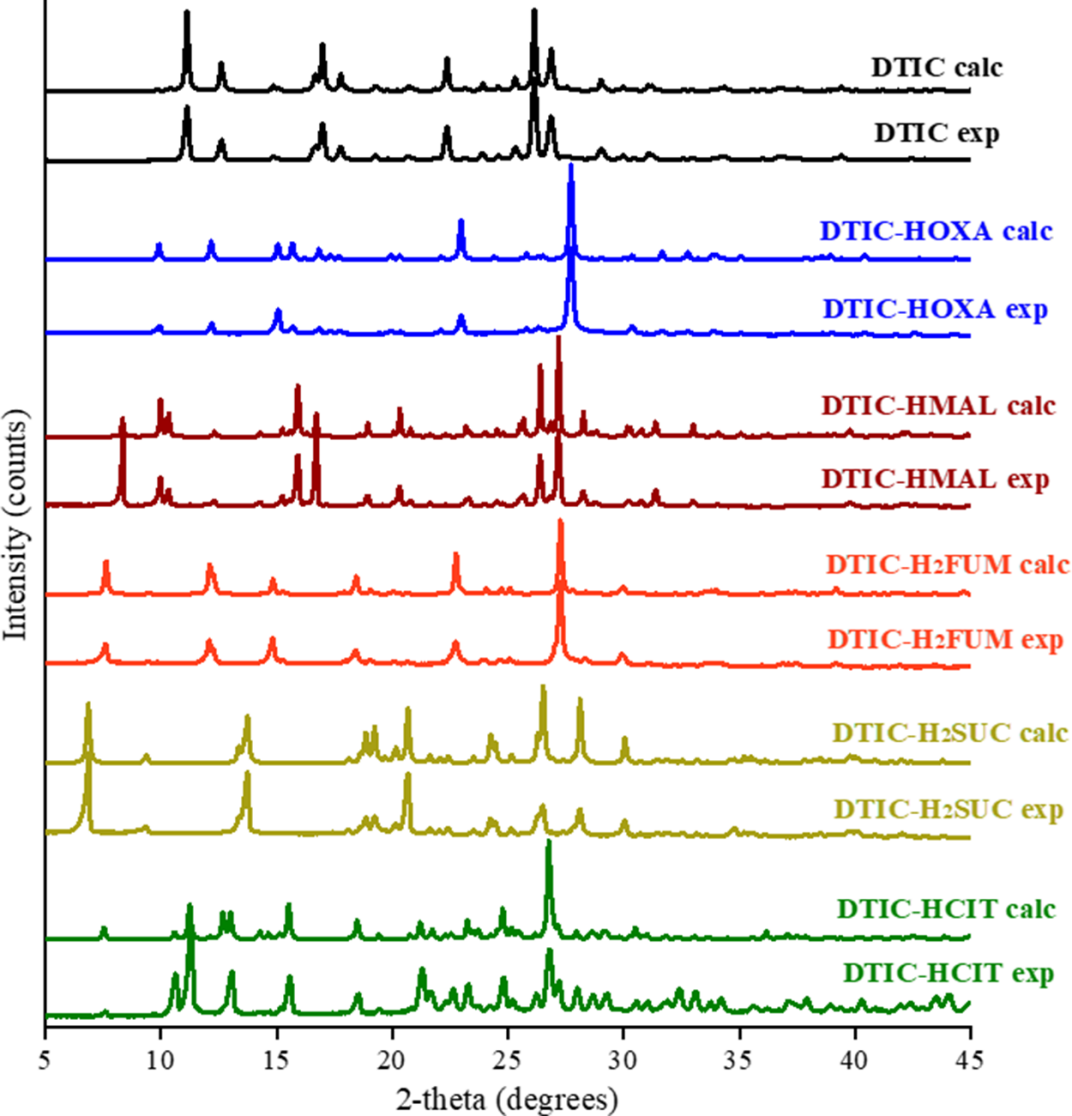
Experimental (exp) and calculated (calcd) diffractograms of DTIC and its new multicomponent crystals.

Powder X-ray diffraction is a powerful solid-state characterization tool to check new multicomponent crystal formation. When we observe that the 2θ peak positions in the experimental diffractograms differ compared to those for starting materials, we conclude that a new and unambiguous crystalline phase has emerged. Additionally, PXRD analysis proves that the single crystal selected for the SCXRD data collection is representative of the whole synthesized sample. As depicted in Figure 3, all experimental PXRD patterns of DTIC crystal forms are distinct concerning pure DTIC. Also, it is noted that the experimental diffractograms exhibit an excellent agreement with the theoretical ones, calculated from the final CIF files generated in SCXRD analyses. Hence, we confirm that all single crystals synthesized correspond to pure and unique crystalline phases and are representative of the entire sample.

### 3.3 Infrared Spectroscopy

Infrared spectroscopy was decisive in confirming the proton transfer and, accordingly, the real position of the hydrogen atoms in the structures. By providing structural information on the molecular vibrational modes, this technique was complementarily used to characterize the new DTIC crystal forms. FT-IR spectra of unmodified DTIC and their multicomponent crystals are shown in Figure S5. For band assignments (Table S6) and spectra interpretation, we rely on the crystallographic description (Section 3.1) and reference spectroscopic data available for DTIC-related compounds.^35,40,41^ DTIC exhibits typical infrared stretching frequencies at 3380 and 3170 cm^–1^ (amide N–H stretches), 1655 cm^–1^ (amide C=O stretch), and 1608 cm^–1^ (C=C stretch). Salt/cocrystal formations have been proved by verification of these characteristic API vibrational modes, mainly due to the emergence of new absorption bands at around 1700 cm^–1^ in the DTIC multicomponent crystal FT-IR spectra. These bands have been attributed to the acid C=O stretching modes of partially deprotonated (oxalate, maleate, and citrate anions) and fully protonated (fumaric and succinic acids) coformers molecules. We also noticed the presence of new weak bands ranging from 1563 to 1405 cm^–1^, attributed to the carboxylate antisymmetric and symmetric stretching modes of the DTIC salt forms. All these considerations follow the previously presented crystallographic evaluations.

### 3.4 Proton Nuclear Magnetic Resonance

The ^1^H NMR experiments certified the stoichiometry of the DTIC:coformer through integral values and further ascertained the sample purity in solution. Assignments of proton chemical shifts and spectra interpretation were performed using DTIC ^1^H NMR spectra reported in the literature.^35,41,42^ Hence, the ^1^H NMR spectra of DTIC salt/cocrystal forms (see Figures S7–S11) displayed the predicted DTIC signals and also the typical signals, e.g., =CH (olefinic) and −CH_2_ (methylene), of the carboxylic acid molecules that are absent in the DTIC ^1^H NMR spectrum (Figure S6). The absence of unassigned proton signals in the ^1^H NMR spectra reinforces the purity of the synthesized crystals. Finally, the integral values found in the salt and cocrystal spectra corroborate that all crystal structures comprise DTIC and the corresponding coformer molecule in the composition established in crystallographic analyses.

### 3.5 Thermal Characterization

**Figure 4:**
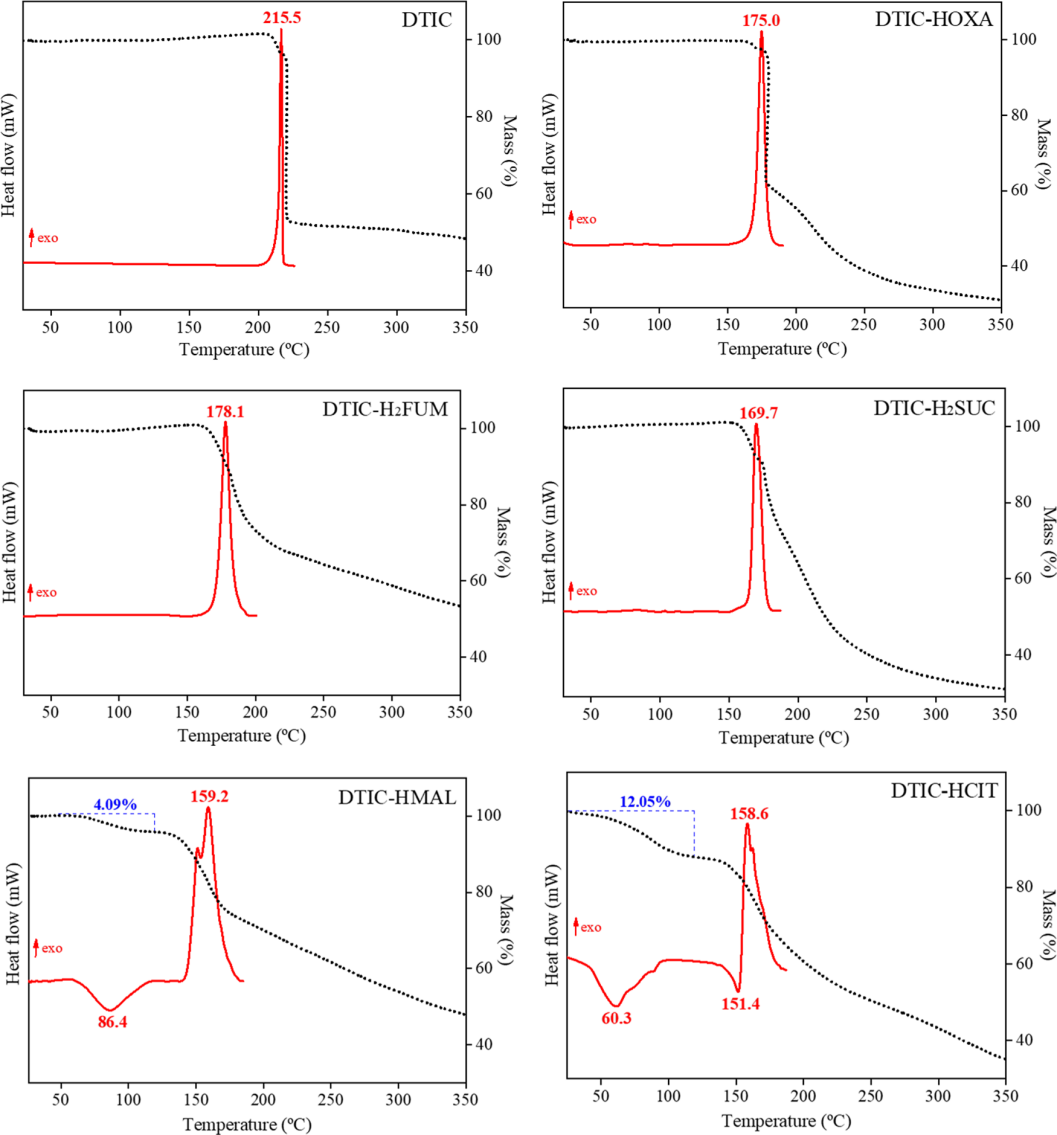
DSC curves (red solid line) and TG thermograms (black dashed line) for the DTIC solid forms.

The thermal behavior and phase purity of the DTIC crystal forms were assessed by DSC and TG, as illustrated in Figure 4. DSC and TG thermograms of pure DTIC were included for comparison. According to the TG curve, DTIC is thermally stable up to 208 °C (*T*_onset_ = 206.5 °C), and its DSC curve shows a single exothermic degradation peak at 215.5 °C (*T*_onset_ = 210.8 °C). Likewise, the DTIC-HOXA DSC curve is also featured by a single degradation exothermic peak centered at 175.0 °C (*T*_onset_ = 170.2 °C). This unique thermal event agrees with the mass loss that occurs in the TG curve, beginning at around 166 °C (*T*_onset_ = 161.7 °C). Following this trend, DSC curves of both DTIC-H_2_FUM and DTIC-H_2_SUC cocrystals are characterized by a unique exothermic peak at 178.1 °C (*T*_onset_ = 172.1 °C) and 169.7 °C (*T*_onset_ = 165.2 °C), respectively, which were attributed to the sample decomposition. These values agree with the gradual mass loss that occurs in the TG curves, which begins at around 163 °C (*T*_onset_ = 159.4 °C) for DTIC-H_2_FUM and at 156 °C (*T*_onset_ = 152.6 °C) for DTIC-H_2_SUC.

For DTIC-HMAL and DTIC-HCIT hydrated salts, both DSC curves displayed early endothermic peaks below 120 °C, corresponding to sample dehydration, i.e., loss of water molecules from the crystalline lattice. These events were followed by initial mass losses on TG curves of about 4.1 and 12.0% in the same temperature interval, consistent with the number of structural water molecules expected for each salt (see Section 3.1). In sequence, the resulting DSC/TG profiles of the dehydrated salt phases become even more similar. Exothermic degradation peaks centered at around 159 °C on DSC curves, accompanied by gradual mass losses on TG thermograms in this referred temperature, summarize the DTIC-HMAL and DTIC-HCIT thermal profiles. The difference between these salts refers to the discrete endothermic melting peak at 151.4 °C, existing only in the citrate salt DSC curve.

In terms of purity, we did not find any degradation traces in the synthesis or crystallization process. All of the crystals obtained were colorless and did not show any light pink spots, which could indicate the presence of 2-azahypoxanthine. The drug purity check by LC (Table 3) and a careful inspection of each chromatogram reinforce that there is no degradation during the preparation of the solid forms. Furthermore, from the DSC curve and PXRD pattern of pure 2-azahypoxanthine (Figure S12), it was concluded that the formation of this photodegradation product did not occur during the preparation of the multicomponent solids. From a pharmaceutical perspective, although the new solids have lower melting points than DTIC, they still undergo fusion or degradation at temperatures higher than those typically used in pharmaceutical processing. Even in hydrated forms, the dehydrated structures remain stable until they melt or degrade at around 150 °C. High temperatures of above 70 °C are not commonly used in the storage and preparation of DTIC drug products. Thus, the thermal profile of the new solid forms is adequate and safe with further processing without any negative impact.

### 3.6 Solubility and Intrinsic Dissolution Profiles

Solubility and dissolution rate are the most important biopharmaceutical attributes of APIs, since they directly impact the pharmacological response.^43^ Enabling the fine-tuning of these properties, particularly for APIs with low and pH-dependent solubility, a crystal engineering strategy has been extensively employed. For neutral DTIC, which exhibits high photodegradation in solution, i.e., cannot remain in solution for a long time, the crystal form synthesis of high solubility is a central requirement for a more efficient pharmaceutical manufacturing process and drug administration. Freeze-dried dacarbazine powder for injection, administered to oncology patients, is prepared by dissolving the API in a solution containing citric acid as an acidifying agent in a step immediately before lyophilization.^44^ At low pH, the faster solubilization of DTIC is followed by its photodegradation.^45^ To alleviate this issue, the API needs to be solubilized at a pH above 4.5, which is unfeasible given the pH-dependent solubility of parent DTIC.^18^ The novel salt-cocrystal forms described herein overcome, in part, this API deficiency.

**Figure 5:**
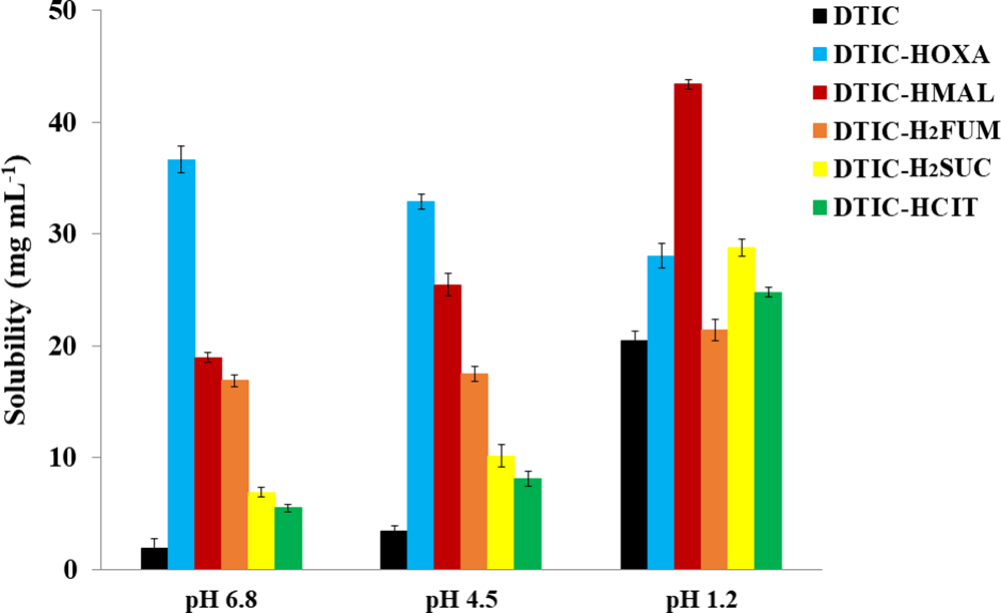
Equilibrium solubility plot of DTIC and its salt and cocrystal forms in different dissolution media.

The equilibrium solubility plot of unmodified DTIC and its new crystal forms in media covering the physiological conditions (HCl solution, pH 1.2; acetate buffer, pH 4.5; and phosphate buffer, pH 6.8) is shown in Figure 5. Overall, considerable enhancements in drug solubility have been reached in all dissolution media evaluated. First, as a typical weak base, DTIC solubility is indeed pH-dependent and agrees with data described in the literature with the highest value in an acidic medium (20.4 ± 0.2 mg mL^–1^). Similarly, the new salt/cocrystal forms showed solubilities superior to 20 mg mL^–1^ in HCl solution at pH 1.2 (see Table 2). In contrast and corroborating our expectations, DTIC-HOXA showed a remarkable solubility improvement above pH 4.5, approximately 20 and 10 times more soluble than pure DTIC in phosphate and acetate buffers, respectively. Also, the other DTIC crystal forms proved to be more soluble over pure APIs at both buffered media, following the solubility order: DTIC-HOXA > DTIC-HMAL > DTIC-H_2_FUM > DTIC-H_2_SUC > DTIC-HCIT > DTIC.

**Table 2:** Equilibrium Solubility Values and Intrinsic Dissolution Rates (IDRs) of the DTIC Solid Forms^a^

crystal form	solubility(mg mL^–1^)			IDR (mg cm^–2^ min^–1^) pH 6.8
	pH 6.8	pH 4.5	pH 1.2	
DTIC	1.9 ± 0.1	3.4 ± 0.2	20.4 ± 0.2	0.26 ± 0.02
DTIC-HOXA	36.6 ± 0.9	32.9 ± 0.3	28.0 ± 0.5	5.58 ± 0.08
DTIC-HMAL	19.0 ± 0.5	25.5 ± 0.7	43.4 ± 0.3	1.90 ± 0.06
DTIC-H_2_FUM	16.9 ± 0.6	17.5 ± 0.6	21.4 ± 0.7	1.31 ± 0.05
DTIC-H_2_SUC	6.9 ± 0.3	10.2 ± 0.7	28.8 ± 0.2	0.43 ± 0.03
DTIC-HCIT	5.5 ± 0.4	8.1 ± 0.2	24.8 ± 0.4	0.35 ± 0.01

a IDR: intrinsic dissolution rate.

**Figure 6:**
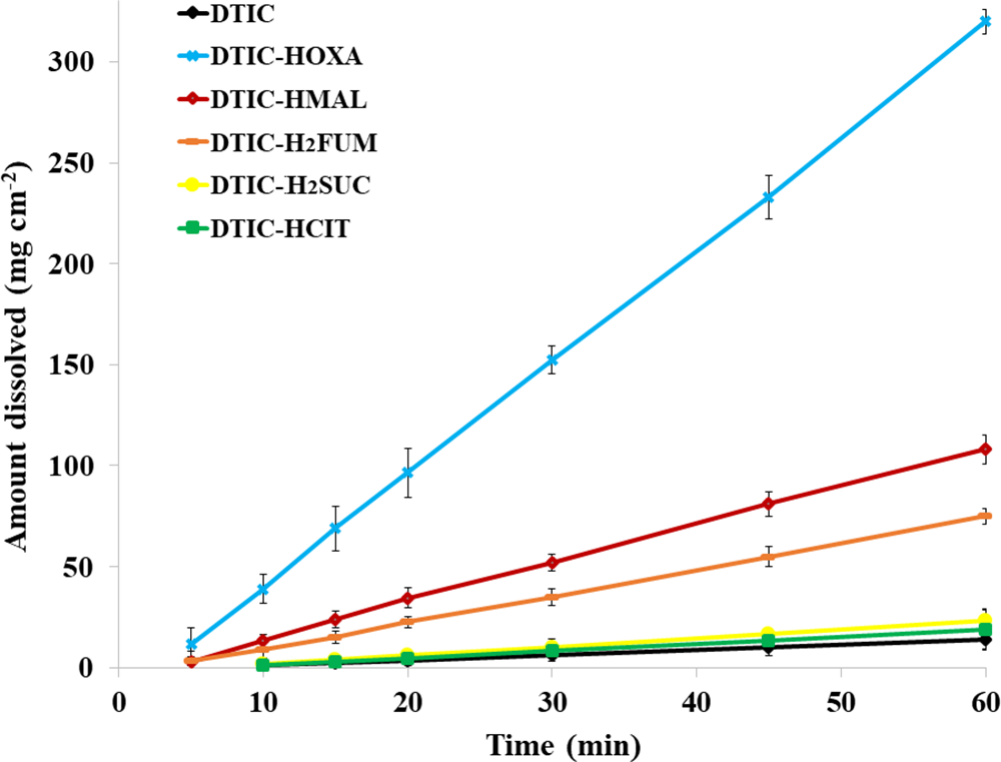
Intrinsic dissolution profile of DTIC and its salt/cocrystal forms in phosphate buffer, pH 6.8.

Regarding the intrinsic dissolution rates (IDRs) measured at phosphate buffer, a medium of less contribution to drug photodegradation, DTIC-HOXA is the fastest dissolving form, displaying a notable IDR of 5.58 ± 0.08 mg cm^–2^ min^–1^. This 22-fold increase compared to the slow IDR of DTIC (0.26 ± 0.02 mg cm^–2^ min^–1^) denotes that oxalate salt tends to be readily solubilized at pH close to 6.8. DTIC-HMAL and DTIC-H_2_FUM further promoted an enhancement of approximately 7.3 and 5.1-fold in the drug dissolution rate, indicating that cocrystallization of DTIC with oxalate, maleic, and fumaric acids has improved the IDR of the API, as depicted in Figure 6. For DTIC-H_2_SUC and DTIC-HCIT, IDR values remained equivalent to those of DTIC. It is essential to mention that all DTIC crystal forms were found to be stable in both solubility and dissolution experiments. The final pH values, measured after the solubility tests, did not display significant variations (Table S8). Moreover, PXRD data showed that the crystal structure of the solid residues and the undissolved disks from the solubility and dissolution studies remained the same as the initial ones (see Figure S13), excluding any evidence of phase transitions.

### 3.7 Stability Outcomes

Besides the encouraging solubility and dissolution findings, the stability examinations of the DTIC crystal forms were quite interesting. Solid-state stability data of freshly synthesized crystals compared to solid samples exposed to accelerated degradation conditions are summarized in Table 3. All initial samples have a maximum purity (DTIC content of ∼100%), and the DTIC samples cocrystallized with carboxylic acids retain almost entirely their native form after 15 days of exposure to UV radiation. The quantity of photoproduct 2-AZA formed was systematically low (not exceeding 2.2%). For pure DTIC, the reduction of 5.6% in the drug content during the photostability test suggests that it is more rational and safer to formulate the API in the form of the new salts or cocrystals of DTIC described in this work. Similar stability outcomes have been found after DTIC solid samples are kept for 90 days at 40 °C and 75% RH, indicating that high temperature and relative humidity have a low degradative potential for the samples.

**Table 3:** DTIC and 2-AZA Levels Found in Freshly Prepared and Accelerated-Degradation DTIC Solid Samples

crystal form	stability (% DTIC and 2-AZA found)					
	freshly prepared (initial)		after 15 days in photostability chamber		after 90 days at 40 °C and 75% RH	
	% DTIC	% 2-AZA^a^	% DTIC	% 2-AZA	% DTIC	% 2-AZA^a^
DTIC	100.0 ± 0.2	-	94.4 ± 0.5	0.8 ± 0.2	99.8 ± 0.3	-
DTIC-HOXA	100.1 ± 0.1	-	97.1 ± 0.3	2.2 ± 0.4	99.9 ± 0.2	-
DTIC-HMAL	99.6 ± 0.4	-	98.4 ± 0.2	1.7 ± 0.2	98.2 ± 0.4	-
DTIC-H_2_FUM	99.5 ± 0.3	-	97.3 ± 0.2	1.1 ± 0.1	99.2 ± 0.2	-
DTIC-H_2_SUC	99.7 ± 0.2	-	98.7 ± 0.3	0.8 ± 0.1	99.3 ± 0.3	-
DTIC-HCIT	99.8 ± 0.4	-	97.0 ± 0.4	1.8 ± 0.2	96.0 ± 0.5	±0.7

a Dashes mean not detected.

Despite the discrete solid-state photostability enhancement of DTIC occasioned by cocrystallization, this contribution has substantial value in the pharmaceutical field. Different from aqueous solubility, which is widely improved by the crystal engineering approach, the generation of photostable APIs is considerably more complex. As already mentioned, the chemical-structural attributes recognized to mitigate the photoinstability of APIs are scarce. According to the literature,^34^ for photolabile drugs, especially photolytic ones, three main strategies are recommended to avoid rapid solid-state photodegradation. First, hindering π–π stacking interactions with appropriate coformers usually makes the drug more photostable. Second, diversifying the occurrence of heterosynthons on sensitive electronic moieties, i.e., preferably subject to photolysis, also retards the API photodegradation. Finally, adopting lattice-spacer coformers that maintain photoreactive species far apart also tends to lower photodegradation.^46^ For all reported DTIC salt and cocrystal forms, the first two strategies are hardly applicable. The π–π stacking interactions are absent in the novel structures, and the sensitive moiety of DTIC, initially subject to photolysis, is not H-bonding interacting with coformers. Thus, the use of spacer coformers seems to be the only feasible strategy to mitigate the photoinstability of DTIC. All of these observations are in line with the discrete drug solid-state photostability enhancement that the salts and cocrystals of DTIC promoted.

**Figure 7:**
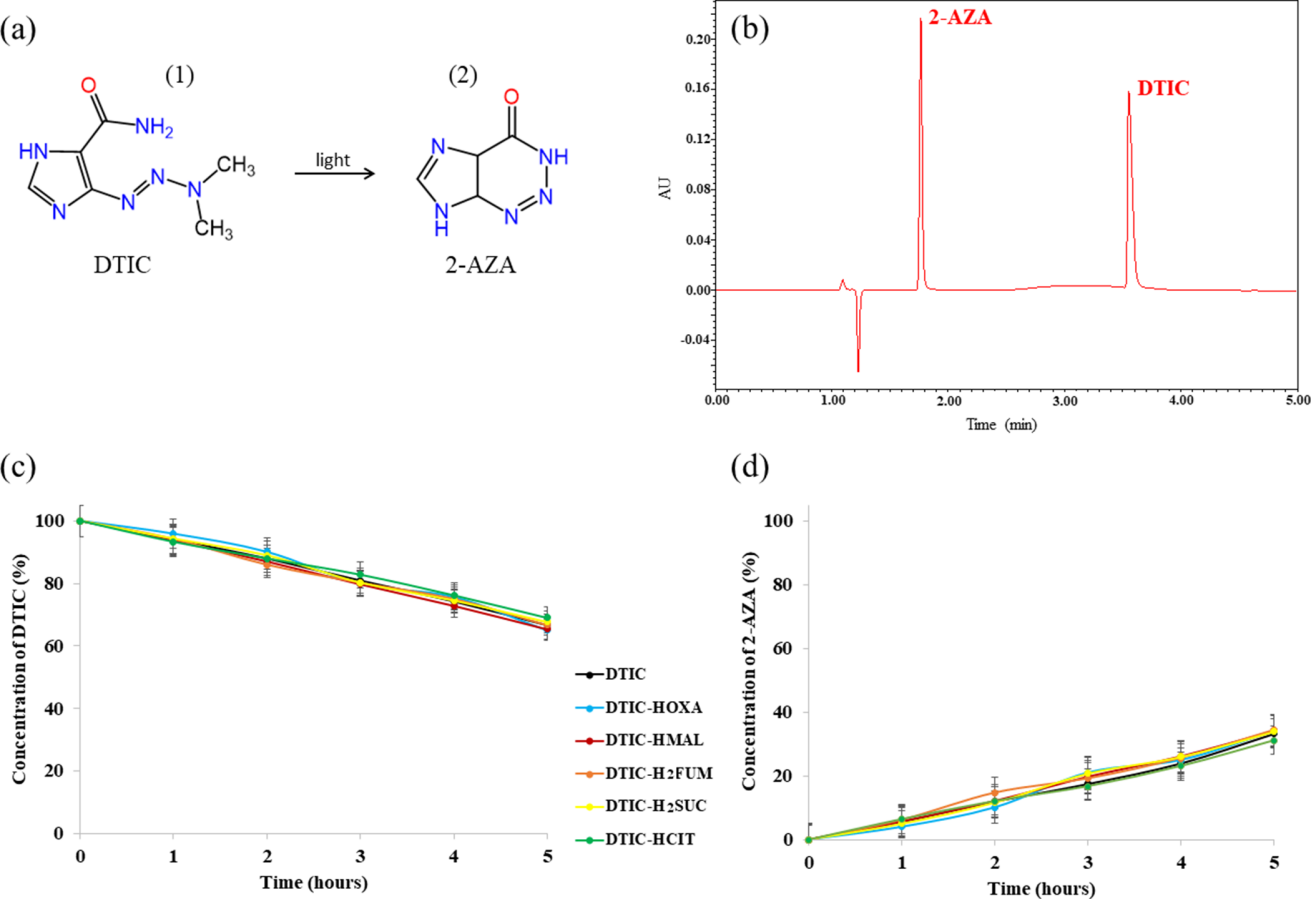
(a) Conversion of DTIC (1) to 2-AZA (2) under light. (b) Chromatogram highlighting the DTIC and 2-AZA separation. Kinetics of (c) DTIC photodegradation and (d) 2-AZA photogeneration from API solutions prepared with each DTIC crystal form.

**Figure 8:**
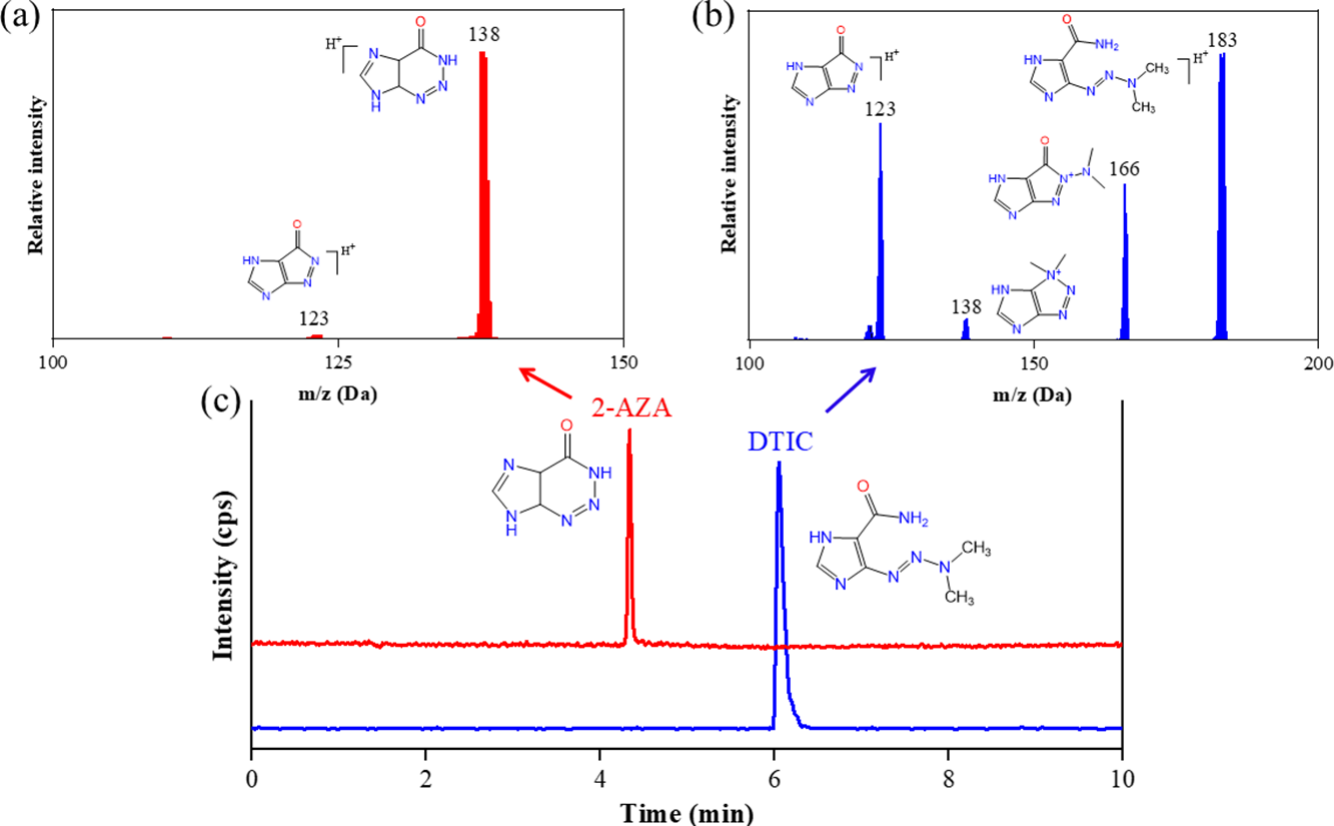
Mass spectrum of (a) 2-AZA and (b) DTIC, showing the main product ions found in fragmentation. (c) Chromatogram of a freshly prepared DTIC sample (blue) compared to the chromatogram of a fully photodegraded DTIC sample (in red).

On the other hand, it was verified that photodegradation from DTIC solutions exposed to UV radiation is quite pronounced, following the literature evidence.^18^ Before conducting photodegradation kinetic studies, we first certified the optimal pH of the solutions and DTIC concentration. Complete and rapid photodegradation of DTIC was observed for low-pH and diluted solutions. Hence, the study had to be conducted at a pH of 6.8 and 5 mM concentration. As illustrated in Figure 7, LC-UV analysis showed only one photoproduct peak in the chromatogram. This peak was confirmed to be 2-AZA since its retention time, at the degraded solution chromatogram, matched with that observed for 2-AZA at the reference standard solution chromatogram (Figure S14). Furthermore, from LC-MS/MS experiments, the mass spectra of DTIC and 2-AZA peaks were obtained. The fragments observed in the DTIC mass spectrum (*m*/*z* 166, 138, and 123) coincided with those reported in the literature.^35^ The 2-AZA mass spectrum displayed only one fragment (*m*/*z* 123), possibly the same as that observed in the DTIC mass spectrum (see Figure 8). Thus, this result strongly suggests 2-AZA as the only DTIC photoproduct under the experimental conditions employed, agreeing with the literature.^18,47^

Regarding the kinetic study, DTIC is converted to 2-AZA with a photodegradation rate constant of 0.756 mM h^–1^. Regardless of whether it is in its parent form or cocrystallized with carboxylic acids, similar DTIC photodegradation and 2-AZA photogeneration rates have been found (Table 4). These observations are more evident when we observe the plots in Figure 7c,d, which correlate these species concentrations as a function of time. The most plausible hypothesis concerning this finding may be related to the absence of photoprotective properties by the aliphatic carboxylic acids used as coformers in solution.

**Table 4:** Photodegradation and Photogeneration Rate Constants of DTIC and 2-AZA for Each DTIC Crystal Form^a^

solution	*k*_d_ (mM h^–1^)	*k*_g_ (mM h^–1^)
DTIC (5 mM)	0.767 ± 0.02	0.361 ± 0.04
DTIC-HOXA (5 mM)	0.756 ± 0.03	0.386 ± 0.03
DTIC-HMAL (5 mM)	0.773 ± 0.01	0.371 ± 0.02
DTIC-H_2_FUM (5 mM)	0.755 ± 0.01	0.363 ± 0.02
DTIC-H_2_SUC (5 mM)	0.747 ± 0.03	0.378 ± 0.05
DTIC-HCIT (5 mM)	0.740 ± 0.02	0.338 ± 0.03

a *k*_d_: photodegradation rate constant for DTIC; *k*_g_: photogeneration rate constant for 2-AZA.

In a series of salts or cocrystals composed of dicarboxylic acids, the carbon chain length of the coformer alternately affects the melting points and solubilities of the solid forms in an odd–even manner.^48^ Generally, dicarboxylic acids with an even number of carbons increase melting points and decrease solubilities compared with those with odd carbons. Structurally, even-chained dicarboxylic acids in *-trans* orientation, such as fumaric and succinic acids, tend to generate more tight crystal packing. On the other hand, odd-chained or *cis*-configurated carboxylic acids, such as maleic acid, exhibit twisted molecular conformations and deviate from linearity compared to their even-chained members, causing more strain when molecules are packed in the crystal. Recent studies have been addressing this topic.^49,50^ Although we have only reported DTIC forms with even acids, we have noted that the structures with the *-trans*/symmetrical fumaric and succinic acids are thermally more stable (Table S7), less soluble, and have slower dissolution (Table 2) than those with the *-cis*/asymmetrical maleic and citric acids, supporting the even–odd concept. The DTIC photodegradation does not appear to be affected by these chemical-structural attributes.

Pragmatically, the new salts and cocrystals of DTIC offer advantages over the original DTIC, making them suitable for the development of a new drug product. Among the solid forms we synthesized, the DTIC oxalate salt stands out for its biopharmaceutical profile. It is considerably more soluble and slightly more photostable in the solid state than DTIC. We anticipate that our future research using these new multicomponent solid forms, focused on enhanced formulations of DTIC, will support these initial findings. In terms of the scale-up of these potential new APIs, the mechanochemistry approach appeared promising, since the use of this technique on a laboratory scale proved to be effective with adequate yield. Additionally, we may consider using seeding-based crystallization experiments as a strategy for scaling up. To mitigate API photodegradation, it is recommended to employ the shortest possible time for maintaining DTIC in water, avoiding the acidic pH range and using a more soluble and faster dissolving DTIC salt or cocrystal instead of pure DTIC, which is less soluble and dissolves more slowly. It is also important to keep all containers in which DTIC is dissolved protected from light and, if possible, at a low temperature.

## 4 Conclusions

Within our efforts to develop improved multicomponent crystals of anticancer drugs, in this work, we reported five novel salt/cocrystal forms of DTIC with pharmaceutically acceptable carboxylic acids (oxalic, maleic, fumaric, succinic, and citric). These crystal modifications were engineered to enhance DTIC solubility and the dissolution rate, making the API less prone to photodegradation in pharmaceutical processes. Analysis of salt/cocrystal structures by SCXRD revealed that 3D packings are layered structures stabilized mainly by H-bonds. The spectroscopic (FT-IR and ^1^H NMR), PXRD, and thermal (DSC and TG) data were congruent with the crystallographic evaluation, confirming pure multicomponent crystal formation. Additionally, in the solubility and dissolution tests, the values found have demonstrated a meaningful increase in these properties, especially for the DTIC-HOXA salt, which displayed a solubility and dissolution rate approximately 20 times higher at pH 6.8 compared to pure DTIC. The DTIC photodegradation and 2-AZA photogeneration in solution, in turn, have not changed regardless of DTIC being cocrystallized or being on its parent form. We have concluded that the cocrystallization of the antineoplastic agent dacarbazine with aliphatic carboxylic acids offers one of the central requirements, i.e., prompt solubilization of the API, to optimize the pharmaceutical product manufacturing process. This investigation also extends the literature on new DTIC solid forms, providing valuable insights into the design of future improved APIs based on DTIC.

## Data Availability

CCDC 2334988–2334992 contains the supplementary crystallographic data for this paper. These data can be obtained free of charge *via*www.ccdc.cam.ac.uk/data_request/cif, or by emailing data_request@ccdc.cam.ac.uk, or by contacting The Cambridge Crystallographic Data Centre, 12 Union Road, Cambridge CB2 1EZ, UK; fax: + 44 1223 336033.

## Supporting Information Available

Additional Tables and Figures from crystal structure analysis (including ASUs), ^1^H NMR spectra, and complementary PXRD patterns of DTIC crystal forms (PDF)
