# Supplementary material for: Highly Soluble Dacarbazine Multicomponent Crystals Less Prone to Photodegradation
Source: Mol Pharm. 2024 Jun 10;21(7):3661–73. doi: 10.1021/acs.molpharmaceut.4c00393 (PMC11220790; doi:10.1021/acs.molpharmaceut.4c00393)
Supplement: Supplementary file 1 — mp4c00393_si_001.pdf [file mp4c00393_si_001.pdf]

ELECTRONIC SUPPLEMENTARY INFORMATION

Highly soluble dacarbazine multicomponent crystals less prone to photodegradation

Luan F. Diniz[a], Paulo S. Carvalho Jr[c], Mateus A. C. Souza[a], Renata Diniz[b], Christian Fernandes[a*]*

[a]Laboratório de Controle de Qualidade de Medicamentos e Cosméticos, Departamento de Produtos Farmacêuticos, Faculdade de Farmácia, Universidade Federal de Minas Gerais, 31270-901, Belo Horizonte, MG, Brazil.

[b]Departamento de Química, Instituto de Ciências Exatas (ICEx), Universidade Federal de Minas Gerais, 31270-901, Belo Horizonte, MG, Brazil.

[c]Instituto de Física, Universidade Federal do Mato Grosso do Sul, 79074-460, Campo Grande, MS, Brazil.

*Corresponding Authors: luandiniz@ufmg.br; cfernandes@farmacia.ufmg.br

COMPLEMENTARY FIGURES AND TABLES

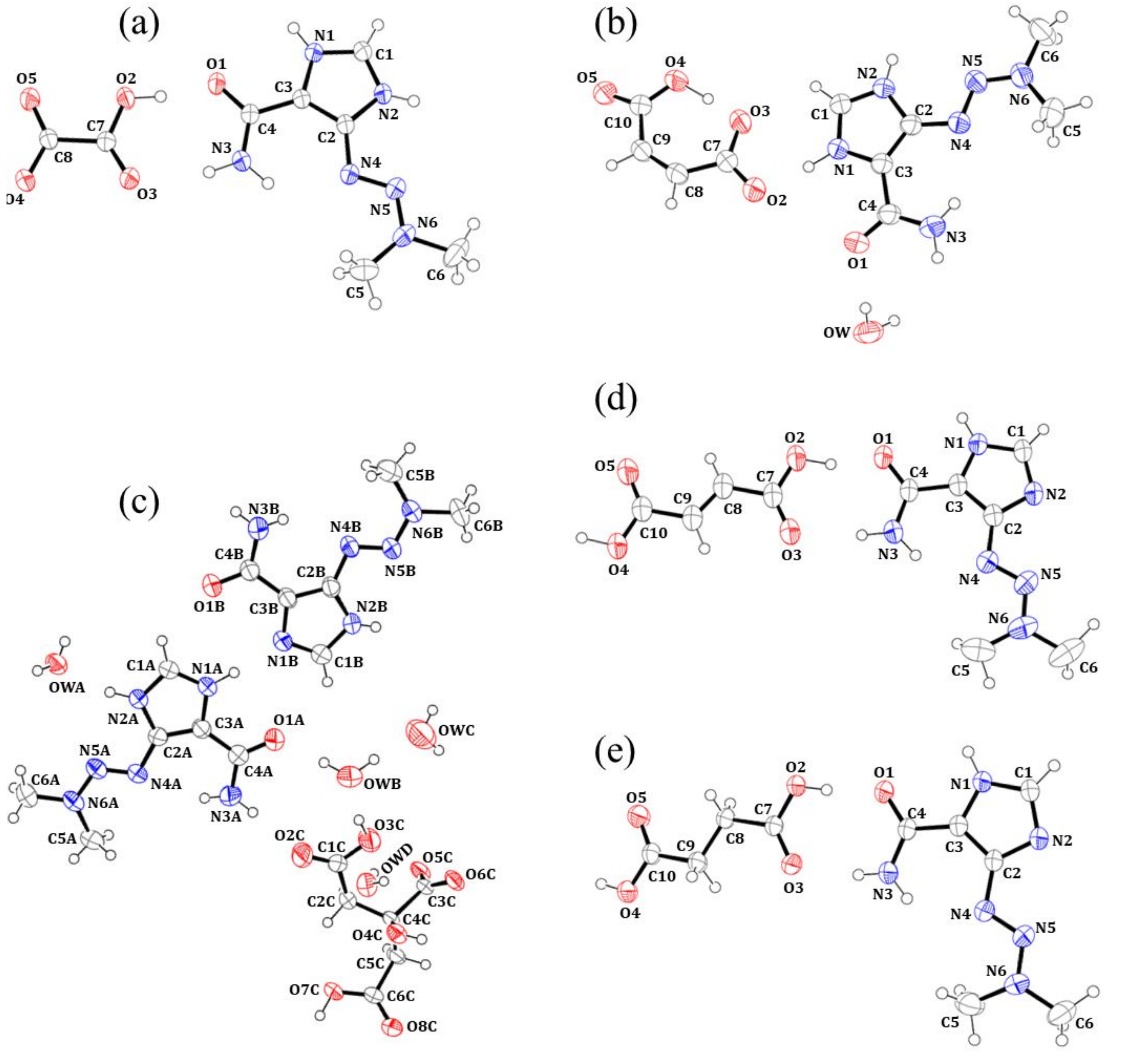

Figure S1. ORTEP type diagrams of the DTIC crystal forms asymmetric units with 50% probability ellipsoids showing atomic numbering scheme: (a) dacarbazine hydrogen oxalate (DTIC-HOXA), (b) dacarbazine hydrogen maleate (DTIC-HMAL), (c) dacarbazine hydrogen citrate (DTIC-H_2CIT), (d) dacarbazine-fumaric acid (DTIC-H_2FUM), and (e) dacarbazine-succinic acid (DTIC-H_2SUC). Hydrogen atoms are shown as spheres of arbitrary radii.

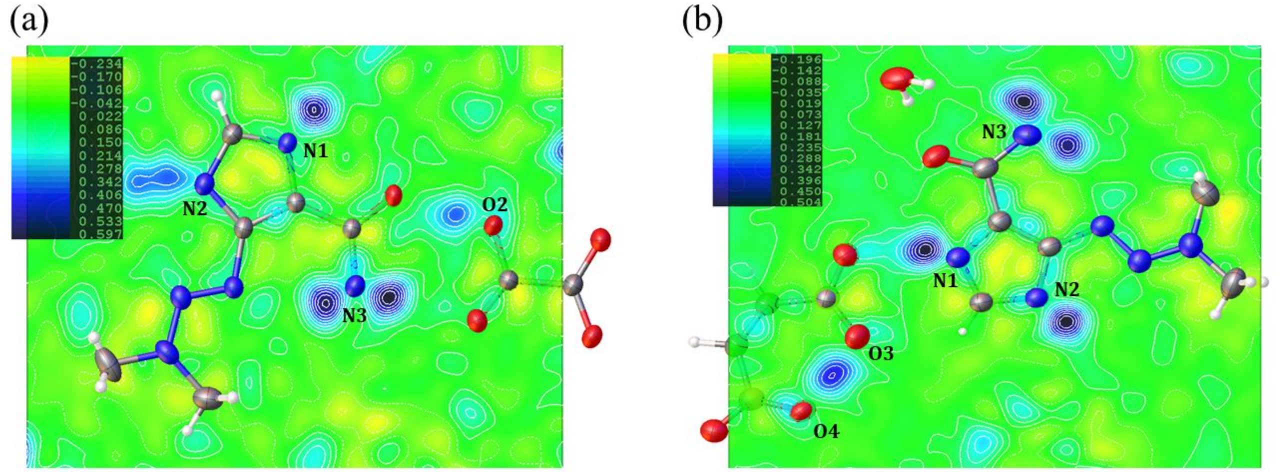

Figure S2. Fourier difference maps, under different perspectives, of dacarbazinum cations ($DTICH^+$) besides both hydrogen oxalate ($HOXA^-$), and hydrogen maleate ($HMAL^-$) anions from the salts (a) DTIC-HOXA and (b) DTIC-HMAL, showing in which O- and N- atoms the H-atoms are attached (blue spots).

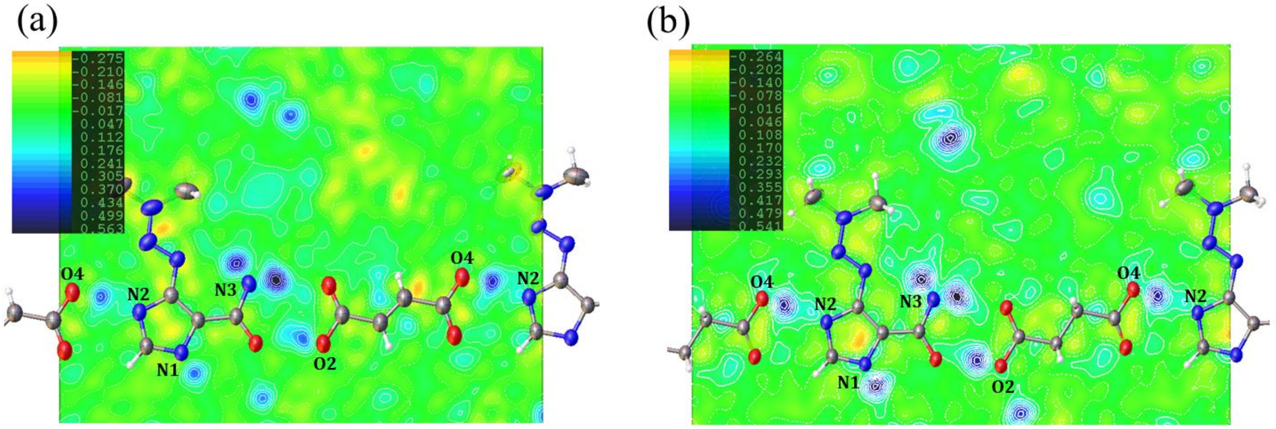

Figure S3. Fourier difference maps, under different perspectives, of dacarbazine molecules (DTIC) besides both fumaric acid (H_2FUM), and succinic acid (H_2SUC) coformers from the cocrystals (a) DTIC-H_2FUM and (b) DTIC-H_2SUC, showing in which O- and N- atoms the H-atoms are attached (blue spots).

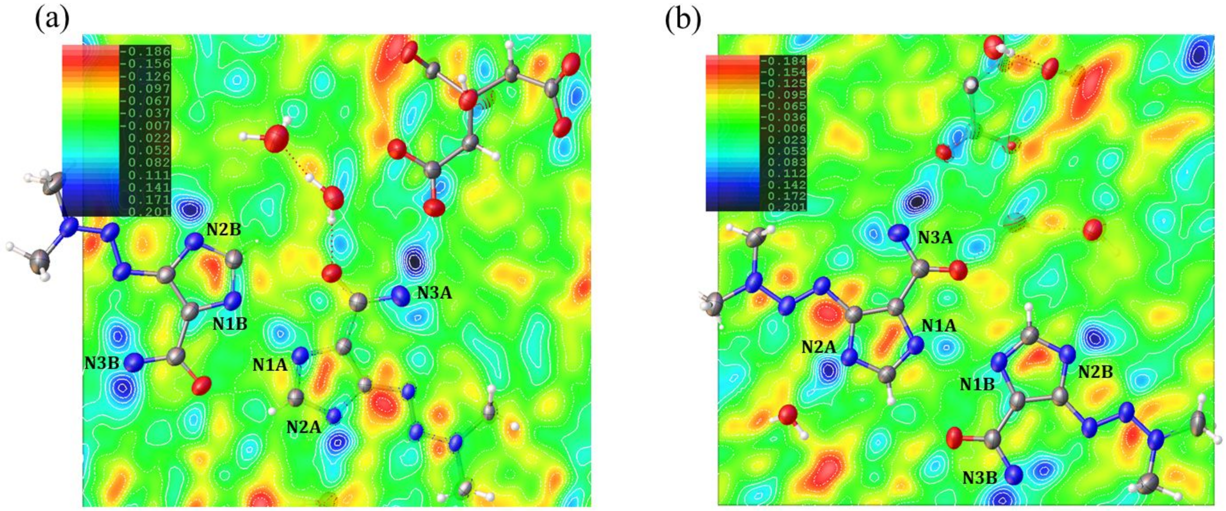

Figure S4. Fourier difference maps, under different perspectives, of dacarbazine (DTIC) and dacarbazinum ($DTICH^+$) molecules from the DTIC-HCIT salt-cocrystal, showing in which N-atoms the H-atoms are attached (blue spots).

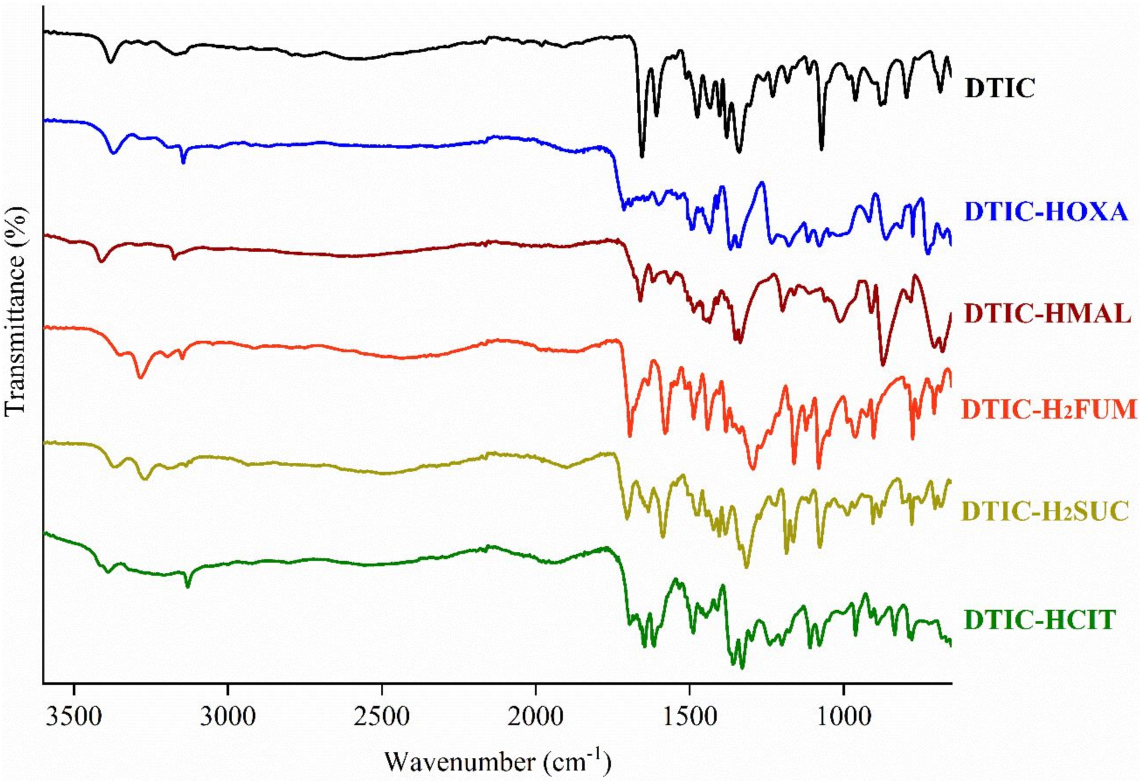

Figure S5. FTIR spectra of DTIC crystal forms.

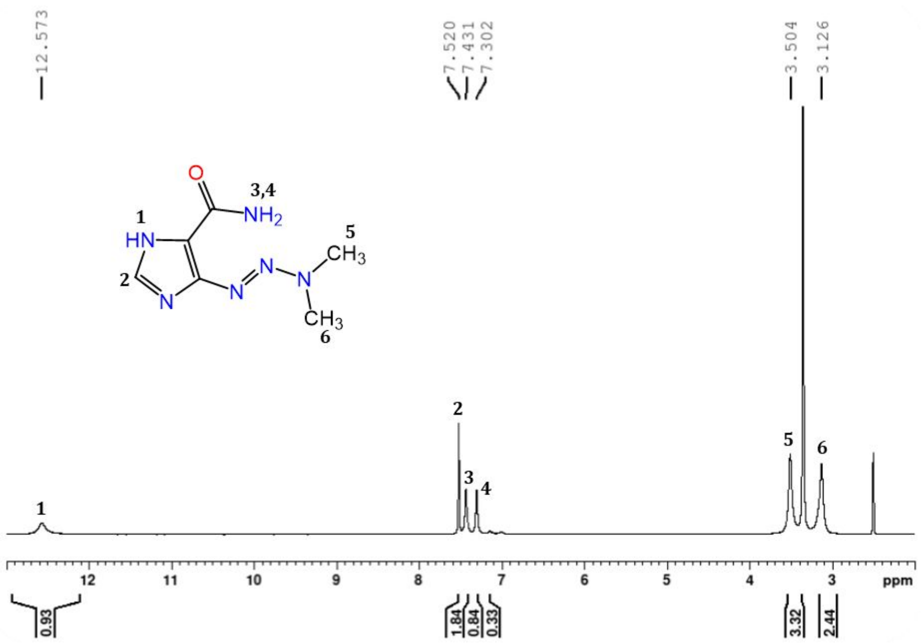

Figure S6. ^{1}H NMR (600 MHz) spectrum of dacarbazine (DTIC) in DMSO-d_6.

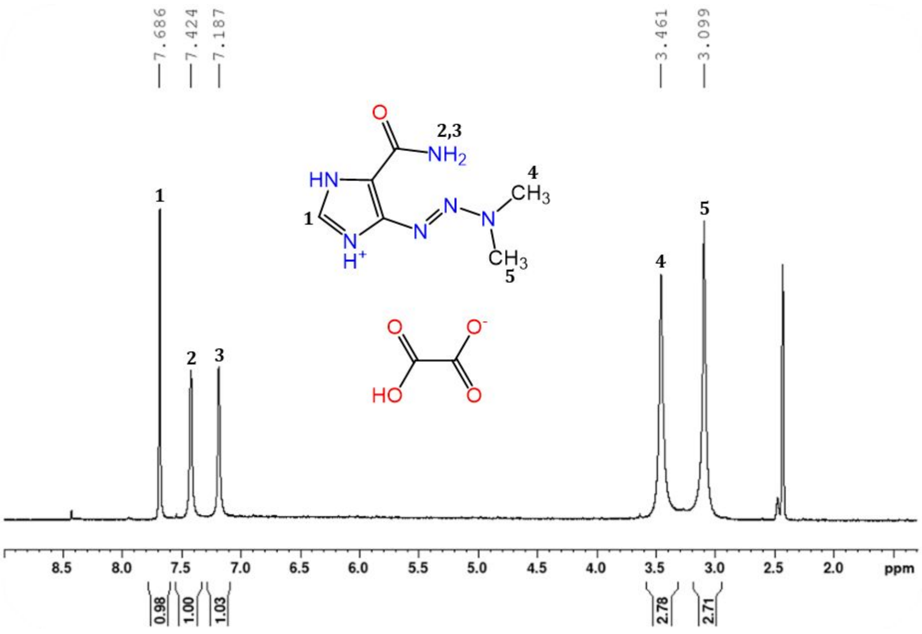

Figure S7. ^{1}H NMR (600 MHz) spectrum of dacarbazine hydrogen oxalate salt (DTIC-HOXA) in DMSO-d_6.

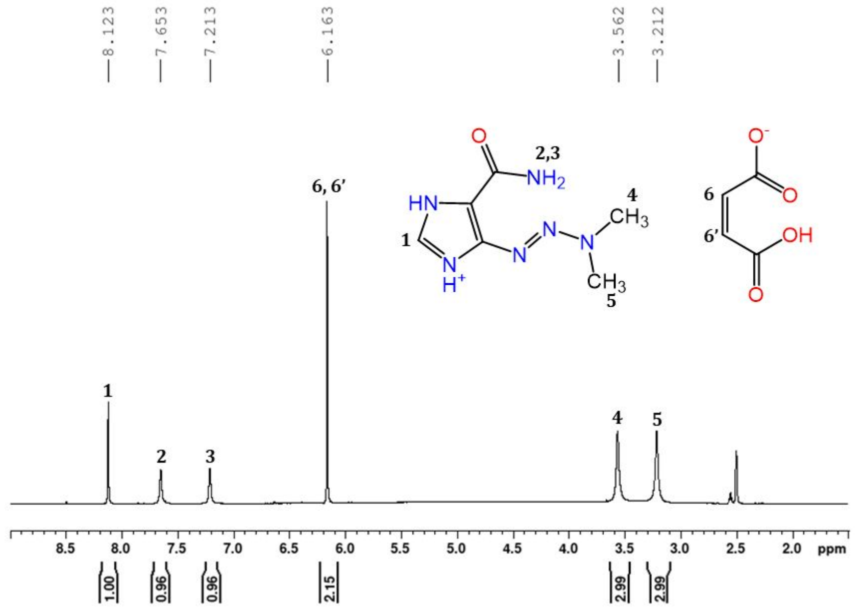

Figure S8. ^{1}H NMR (600 MHz) spectrum of dacarbazine hydrogen maleate salt (DTIC-HMAL) in DMSO-*d*$_6$.

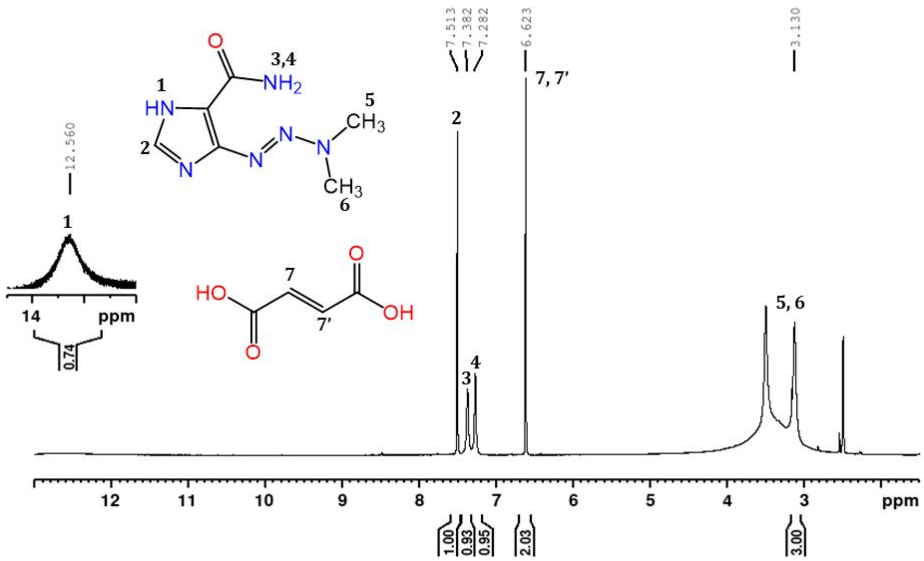

Figure S9. ^{1}H NMR (600 MHz) spectrum of dacarbazine-fumaric acid cocrystal (DTIC-H_2FUM) in DMSO-*d*$_6$.

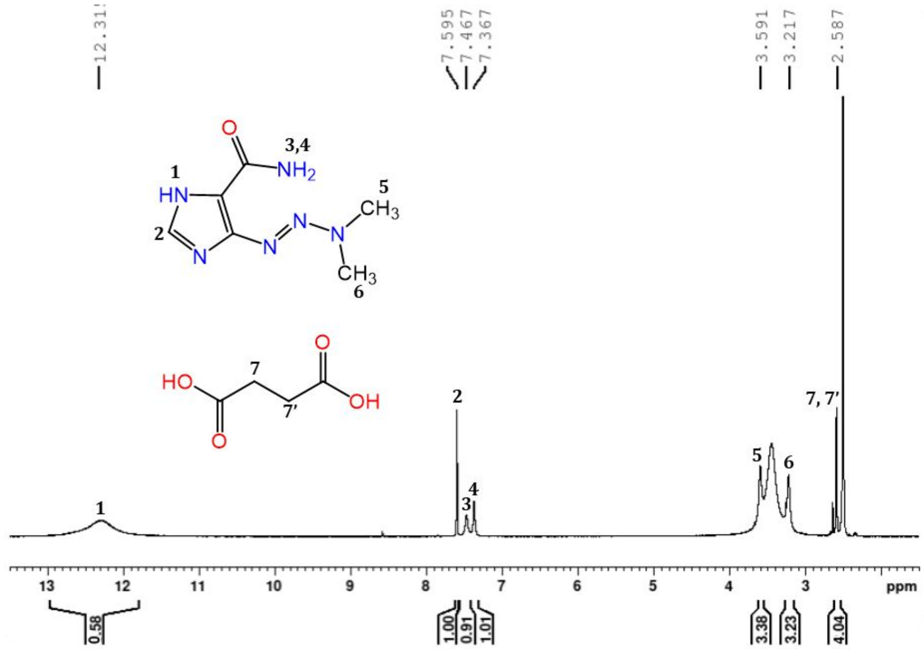

Figure S10. ^{1}H NMR (600 MHz) spectrum of dacarbazine-succinic acid cocrystal (DTIC-H_2SUC) in DMSO-d_6.

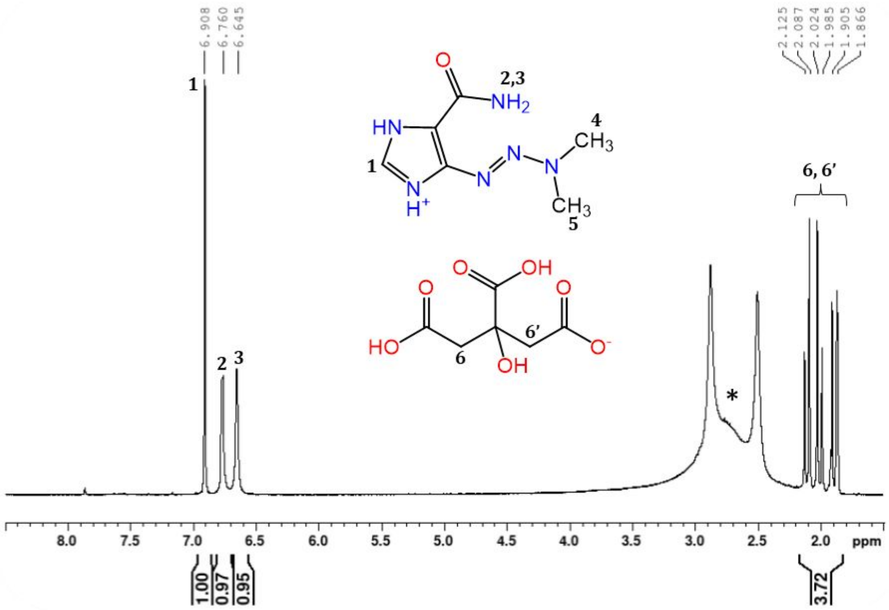

Figure S11. ^{1}H NMR (600 MHz) spectrum of dacarbazine hydrogen citrate salt-cocrystal (DTIC-HCIT) in DMSO-d_6. * Represents the signals of DMSO and H_2O (solvents) as well as the signal of crystallization water molecules (crystal structure).

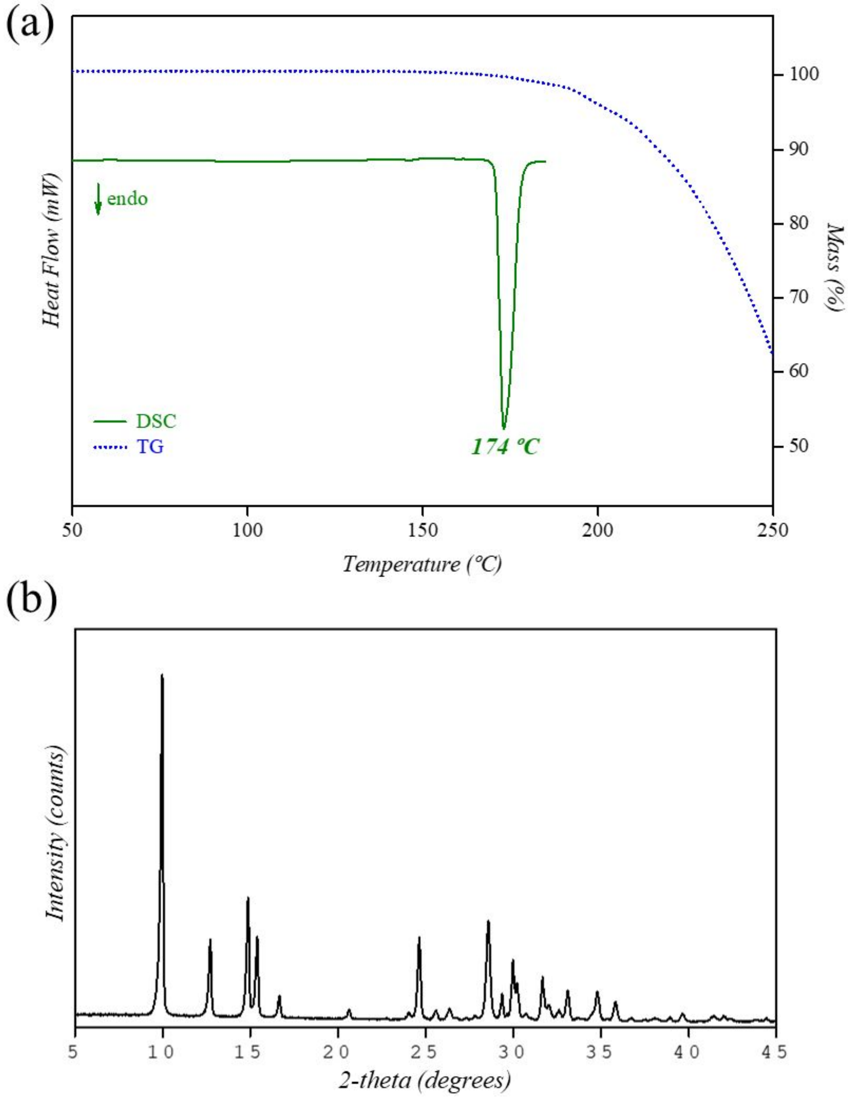

Figure S12. (a) Thermal (DSC and TG) and (b) PXRD data of 2-AZA.

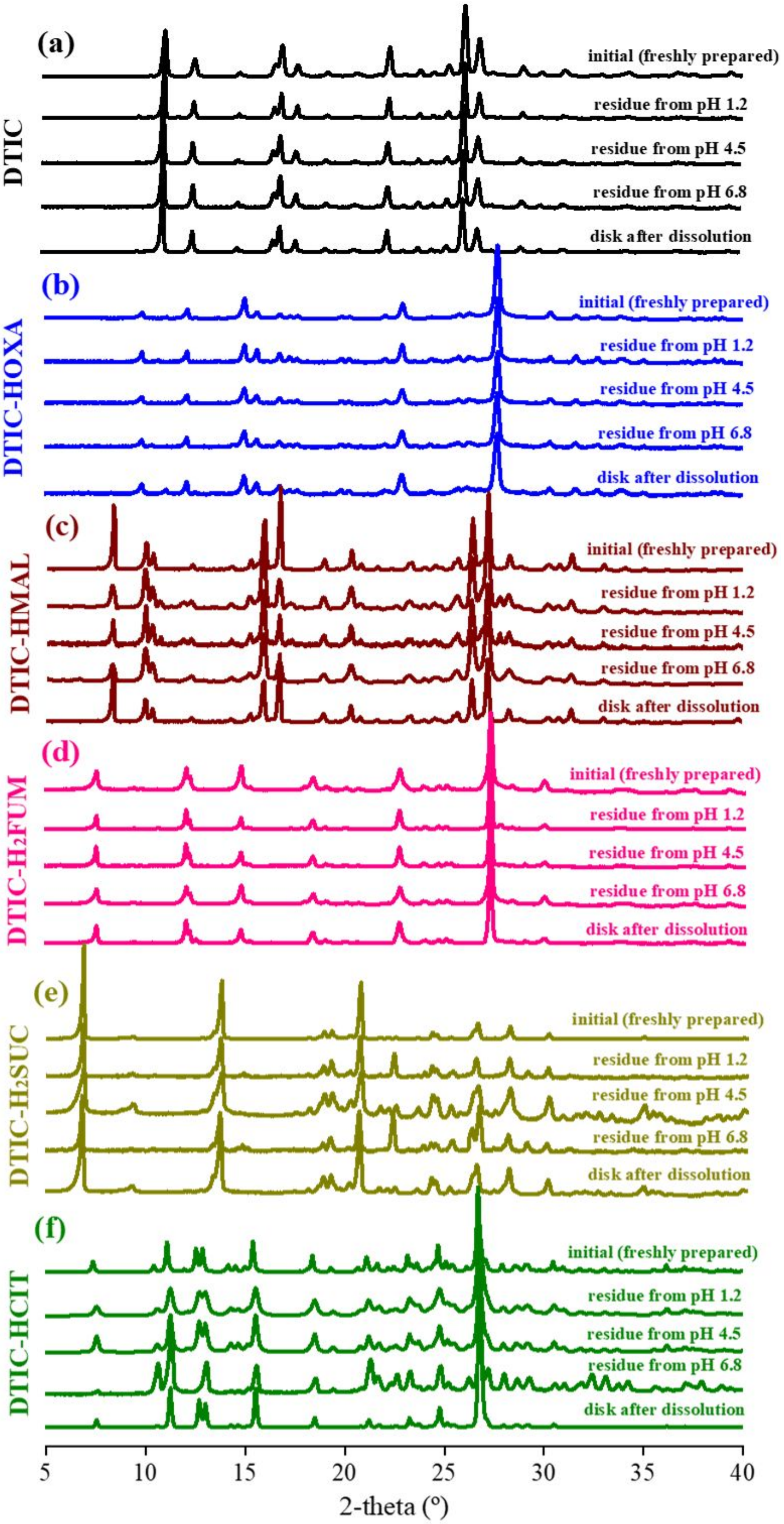

Figure S13. PXRD data of the solid residues and remaining disks from the solubility and dissolution studies for (a) DTIC, (b) DTIC-HOXA, (c) DTIC-HMAL, (d) DTIC-H_2FUM, (e) DTIC-H_2SUC, and (f) DTIC-HCIT.

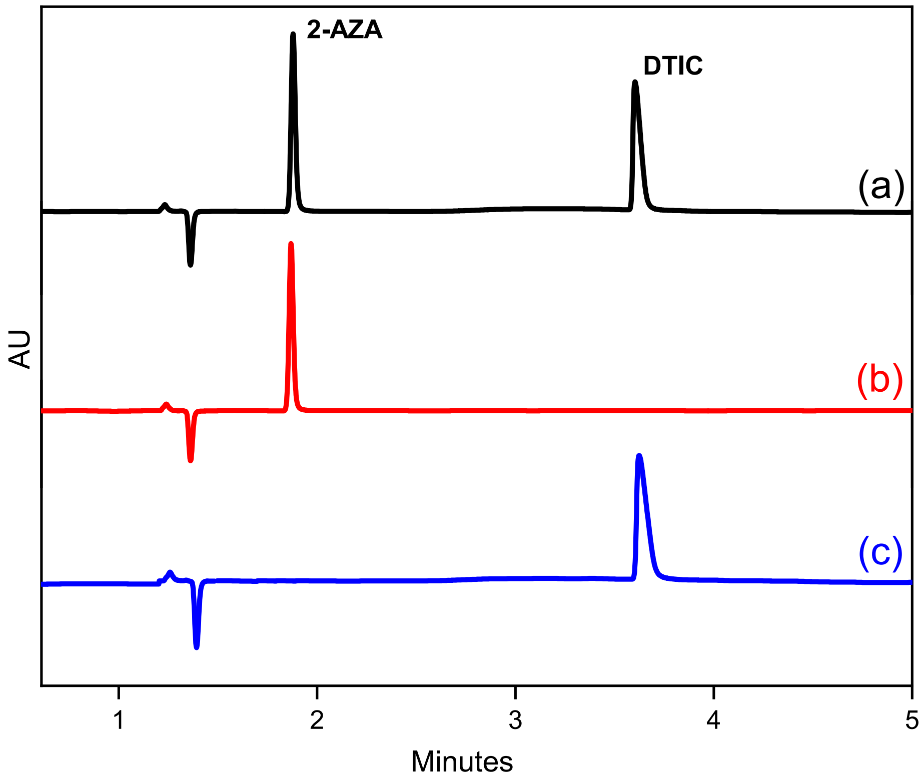

Figure S14. (a) Typical chromatogram of dacarbazine (DTIC) solution UV irradiated, showing the 2-AZA (photoproduct) and DTIC peaks. Chromatograms of authentic (b) 2-AZA and (c) DTIC standard solutions.

Table S1. Results of linearity, accuracy, precision, and limits of quantitation and detection obtained during analytical method validation for DTIC.

Parameter	Results
	Linearity
Concentration range (μg mL^{-1})	1.0-9.0
Slope (b)	1796.3
Intercept (a)	1149.9
Coefficient of determination (R^2)	0.9995
	Accuracy
Level 1 (1.0 μg mL^{-1})	Mean recovery: 100.93% (n = 6)
Level 2 (5.0 μg mL^{-1})	Mean recovery: 101.03% (n = 6)
Level 3 (9.0 μg mL^{-1})	Mean recovery: 99.06%(n = 6)
	Precision
Repeatability (RSD%)	0.56-1.67 (n = 3)
Intermediate (RSD%)	0.40-1.38 (n = 6)
	Limits
Quantitation (μg mL^{-1})	0.12
Detection (μg mL^{-1})	0.04

RSD, relative standard deviation.

Table S2. Results of linearity, accuracy, precision, and limits of quantitation and detection obtained during analytical method validation for 2-AZA.

Parameter	Results
	Linearity
Concentration range (μg mL^{-1})	1.0-9.0
Slope (b)	14219.3
Intercept (a)	-1043.9
Coefficient of determination (R^2)	0.9999
	Accuracy
Level 1 (1.0 μg mL^{-1})	Mean recovery: 101.8% (n = 6)
Level 2 (5.0 μg mL^{-1})	Mean recovery: 99.2% (n = 6)
Level 3 (9.0 μg mL^{-1})	Mean recovery: 100.3% (n = 6)
	Precision
Repeatability (RSD%)	0.70-1.21 (n = 3)
Intermediate (RSD%)	0.80-1.05 (n = 6)
	Limits
Quantitation (μg mL^{-1})	0.06
Detection (μg mL^{-1})	0.02

RSD, relative standard deviation.

Table S3. Composition of the solutions and standard buffer solutions used in the solubility and dissolution experiments.

**Hydrochloric Acid (HCl) Solution (1000 mL)**	
pH	1.2
Conc. (mol L^{-1})	0.1
$HCl_{conc.}$ (mL)	8.4
Deionized water to complete the 1000 mL volumetric flask	
**Acetic Acid Solution (1000 mL)**	
Conc. (mol L^{-1})	0.2
$CH_3COOH_{glacial}$ (mL)	11.5
Deionized water to complete the 1000 mL volumetric flask	
**Sodium Acetate Solution (1000 mL)**	
Conc. (mol L^{-1})	0.2
$C_2H_3O_2Na$ (g)	16.4
Deionized water to complete the 1000 mL volumetric flask	
**Acetate Buffer (1000 mL)**	
pH	4.5
0.2 M $C_2H_3O_2Na$ (mL)	220
0.2 M CH_3COOH (mL)	250
Deionized water to complete the 1000 mL volumetric flask	
**Monopotassium Phosphate Solution (1000 mL)**	
Conc. (mol L^{-1})	0.2
KH_2PO_4 (g)	27.2
Deionized water to complete the 1000 mL volumetric flask	
**Dipotassium Phosphate Solution (1000 mL)**	
Conc. (mol L^{-1})	0.2
K_2HPO_4 (g)	34.8
Deionized water to complete the 1000 mL volumetric flask	
**Phosphate Buffer (1000 mL)**	
pH	6.8
0.2 M KH_2PO_4 (mL)	255
0.2 M K_2HPO_4 (mL)	245
Deionized water to complete the 1000 mL volumetric flask	

Table S4. Calculated ΔpKa between DTIC and the salt formers.

Compound	pKa	ΔpKa = (pKa(base) − pKa(acid))	Stoichiometry (DTIC:coformer)
Dacarbazine	4.4	-	-
Oxalic acid	1.2	3.2	1:1 salt
Maleic acid	1.9	2.5	1:1 salt
Fumaric acid	3.0	1.4	1:1 cocrystal
Succinic acid	4.2	0.2	1:1 cocrystal
Citric acid	3.1	1.3	2:1 salt-cocrystal

Table S5. Geometric parameters of the principal H-bonds in the DTIC crystal forms.

Interaction	D···A(Å)	D–H···A(º)	Symmetry Code
		**DTIC-HOXA**	
N3–H3A···O3	3.022(2)	165	*x,y,z*
O2–H2A···O1	2.488(2)	171	*x,y,z*
N2–H2···O4	2.595(1)	174	*x-1,+y,+z-1*
N1–H1···O5	2.938(2)	171	*x-1/2,-y+1/2,+z-1/2*
C5–H5B···O3	3.337(2)	172	*x-1/2,-y+1/2+1,+z-1/2*
C6–H6A···O5	3.478(3)	150	*-x+1/2+1,+y+1/2,-z+1/2*
		**DTIC-HMAL**	
N1–H1···O2	2.703(1)	171	*x,y,z*
Ow–HwA···O1	2.808(1)	164	*x,y,z*
N2–H2···O5	2.812(2)	166	*-x+1,-y+2,-z+1*
N3–H3B···Ow	2.927(2)	171	*-x,-y+1,-z+2*
Ow–HwB···O5	3.093(1)	163	*x-1,+y-1,+z*
C9–H9···O1	3.137(2)	125	*-x+1,-y+2,-z+2*
C6–H6B···O2	3.456(2)	151	*-x,-y+1,-z+1*
		**DTIC-H_2FUM**	
N3–H3A···O3	3.022(3)	172	*x,y,z*
O2–H2···O1	2.565(2)	165	*x,y,z*
N1–H1···O1	2.921(2)	155	*-x,-y+1,-z+1*
O4–H4···N2	2.645(2)	174	*x,+y,+z-1*
C1–H1A···O2	3.159(2)	136	*-x,+y,-z+1*
C1–H1A···O5	3.098(2)	124	*-x,-y+1,-z+1*
C5–H5A···O3	3.582(4)	154	*-x+1,-y+1,-z+1*
C6–H6A···O1	3.588(1)	159	*x+1/2,+y+1/2,+z+1/2*
		**DTIC-H_2SUC**	
O2–H2A···O1	2.587(2)	175	*x,y,z*
N3–H3B···O3	2.903(2)	167	*x,y,z*
N2–H2···O2	2.960(2)	171	*-x+1,-y,-z+1*
O4–H4···N1	2.700(2)	176	*x+1,+y+2,+z*
C1–H1···O5	3.071(2)	120	*x-1,+y-2,+z*
C5–H5C···O3	3.500(2)	149	*-x+1/2,+y-1/2,-z+1/2+1*
C6–H6C···O3	3.318(3)	131	*-x+1/2,+y-1/2,-z+1/2+1*
C8–H8B···O1	3.557(3)	163	*-x+1,-y+1,-z+1*
		**DTIC-HCIT**	
N1A–H1A···N1B	2.743(4)	174	*x,y,z*
N2A–H2A···OwA	2.744(4)	166	*x,y,z*
N3A–H3AA···O2C	2.990(6)	174	*x,y,z*
C1A–H1AA···O1B	2.977(3)	127	*x,y,z*
C1A–H1AA···O6C	3.126(4)	145	*-x+1,+y+1/2,-z+1*
N3B–H3BA···O8C	2.925(5)	158	*-x+1,+y+1/2,-z+1*
N2B–H2B···OwD	2.881(5)	174	*x+1,+y,+z+1*
C1B–H1B···O1A	3.113(4)	125	*x,y,z*
C5B–H5BC···O1A	3.557(7)	157	*x+1,+y,+z+1*
C6B–H6BC···O7C	3.213(7)	119	*x+2,+y,+z+1*
O7C–H7C···O5C	2.591(3)	167	*x-1,+y,+z*
O3C–H3C···OwB	2.588(5)	168	*x,y,z*

C2C–H2CA···OwD	3.574(5)	152	*x,y,z*
OwA–HwAB···O6C	2.812(3)	163	*-x+1,+y+1/2,-z+1*
OwA–HwAA···O4C	2.769(4)	157	*-x,+y+1/2,-z+1*
OwB–HwBA···OwC	2.686(6)	162	*x,y,z*
OwB–HwBB···O1A	2.702(5)	163	*x,y,z*
OwC–HwCB···OwA	2.901(7)	159	*-x+1,+y-1/2,-z+1*
OwC–HwCA···OwD	2.903(5)	145	*x+1,+y,+z+1*
OwD–HwDA···O5C	2.738(4)	170	*x,y,z*
OwD–HwDB···OwB	2.927(5)	155	*x,+y,+z-1*

Table S6. Principal FT-IR bands (cm^{-1}) for the novel DTIC crystal forms.

DTIC	DTIC-HOXA	DTIC-HMAL	DTIC-H_2FUM	DTIC-H_2SUC	DTIC-HCIT	Assignment
3380, 3170	3376, 3145	3411, 3173	3357, 3285	3373, 3275	3414, 3131	$\nu(NH_2)_{amide}$
-	1714	1681	1695	1703	1695	$\nu(CO)_{acid}$
1655	1653	1660	1636	1635	1646	$\nu(CO)_{amide}$
1608	1601	1620	1582	1588	1615	$\nu(C{=}C)$
-	1540	1563	-	-	1535	$\nu_a(COO)_{carboxylate}$
-	1410	1405	-	-	1409	$\nu_s(COO)_{carboxylate}$

v = stretching; *a* = antisymmetric; *s* = symmetric.

Table S7. Main thermal data from the DSC and TG curves of DTIC samples.

**Compound**	Dehydration (ºC)	Melting point (ºC)	Decomposition temperature (ºC)
**DTIC**	-	-	215.5
**DTIC-HOXA**	-	-	175.0
**DTIC-HMAL**	86.4	-	159.2
**DTIC-H_2FUM**	-	-	178.1
**DTIC-H_2SUC**	-	-	169.7
**DTIC-HCIT**	60.3	151.4	158.6

Table S8. pH measured in different dissolution media before and after solubility studies.

Dissolution media	pH 6.8	pH 4.5	pH 1.2
pH (dissolution media before the tests)	6.8	4.5	1.2
pH (DTIC solution)	6.7	4.4	3.2*
pH (DTIC-HOXA solution)	6.7	4.4	1.3
pH (DTIC-HMAL solution)	6.6	4.5	1.3
pH (DTIC-H_2FUM solution)	6.8	4.5	1.2
pH (DTIC-H_2SUC solution)	6.8	4.4	1.2
pH (DTIC-HCIT solution)	6.6	4.4	1.3

* Under acidic conditions, neutral DTIC is readily protonated, raising the pH.
